# Rewiring tumor visibility: The immunopeptidome as a dynamic interface between antigen processing, microenvironmental stress, and immune recognition

**DOI:** 10.3389/fonc.2025.1691719

**Published:** 2025-11-10

**Authors:** Kangkang Zhao, Yunlan Huang, Linlin Chang, Baiyu Wang, Mingshi Ye, Jinhong Qi

**Affiliations:** 1 Department of 4th Radiotherapy, Jilin Cancer Hospital, Changchun, China; 2 Department of 2nd Gynecologic Oncology, Jilin Cancer Hospital, Changchun, China; 3 Department of Anesthesiology, Jilin Provincial Cancer Hospital, Changchun, China; 4 Department of 1st Breast Surgery, Jilin Cancer Hospital, Changchun, China

**Keywords:** tumor immunopeptidome, antigen processing, immunoproteasome, post-translational modifications, immunotherapy biomarkers

## Abstract

The tumor immunopeptidome dictates whether malignant cells remain visible or invisible to immune surveillance, yet its regulation extends far beyond canonical antigen processing. Here, we synthesize recent insights into how proteasomes, immunoproteasomes, transporter associated with antigen processing (TAP), endoplasmic reticulum aminopeptidase (ERAP), and alternative pathways collectively shape peptide presentation, and how tumor-intrinsic rewiring intersects with microenvironmental stressors such as hypoxia, acidity, and epithelial–mesenchymal transition (EMT). We highlight post-translationally modified ligands as a qualitatively distinct class of tumor antigens, expanding the therapeutic landscape. Across various cancers, the immunoproteasome emerges as both a biomarker and a barometer, with prognostic and predictive value contingent upon the immune context. This duality highlights the necessity for context-aware therapeutic strategies, encompassing selective immunoproteasome modulation, TAP2-based biomarkers, and post-translational modification (PTM)-directed vaccines. Framing the immunopeptidome as a dynamic and rewritable interface provides both mechanistic insight into immune escape and a roadmap for precision immuno-oncology.

## 1 Introduction

Cancer remains one of the leading causes of morbidity and mortality worldwide, despite remarkable advances in surgery, chemotherapy, radiotherapy, and targeted therapies (1–4). Tumor heterogeneity and immune evasion are among the significant challenges (5–8) that limit the efficacy and durability of even the most advanced immunotherapies. Understanding how malignant cells become either visible or invisible to the immune system is therefore a fundamental priority in oncology. At the heart of immune recognition is the immunopeptidome, the repertoire of peptides displayed on major histocompatibility complex (MHC) molecules (9, 10). Beyond the classical proteasome–transporter associated with antigen processing (TAP)–endoplasmic reticulum (ER)–MHC-I cascade, regulation of the immunopeptidome is initiated at earlier genomic and transcriptomic levels (11–13). Transcription factors such as signal transducer and activator of transcription 1 (STAT1), interferon Regulatory Factor 1 (IRF-1), nuclear factor kappa B (NF-κB), and hypoxia-inducible Factor 1α (HIF-1α) directly control the expression of antigen-processing machinery (APM) components, including TAP (14, 15), Endoplasmic reticulum aminopeptidase (ERAP) (16–18), and MHC-I molecules, thereby determining the basal readiness of tumor cells for immune recognition. In addition, higher-order chromatin organization and three-dimensional nuclear architecture impose critical constraints on gene accessibility and transcriptional regulation (19–21). Meanwhile, epigenetic modifications, such as DNA methylation and histone acetylation, dynamically tune the transcriptional output of APM genes (22–25). These upstream layers establish a genomic–epigenomic–transcriptomic foundation that precedes peptide processing and frames antigen presentation as a multilevel pathway extending from chromatin regulation to HLA presentation. This repertoire is primarily shaped by classical antigen processing (26, 27), whereby intracellular proteins are degraded by proteasomes (28–30), chaperoned by TAP into the ER (31, 32), subjected to ERAP1/2–mediated trimming (33–35), and finally loaded onto MHC-I molecules for presentation to CD8^+^ T cells (36, 37). This process renders the immunopeptidome responsible for the density, diversity, and specificity of antigenic targets that direct immune surveillance. Importantly, ERAP1/2 do not only edit the non-classical human histocompatibility leukocyte antigen (HLA)-E (HLA-E) ligandome (38); they also calibrate classical HLA-I (HLA-A, -B, -C) peptide pools (39–41).

Recent evidence demonstrates that the tumor immunopeptidome is not a static entity but rather a highly dynamic and context-dependent system, continuously reshaped by both tumor-intrinsic and microenvironmental cues. Multifactorial drivers such as cytokine signaling and therapeutic stresses are reported to reorganize the antigenic cancer cell landscape, altering the diversity and prevalence of presented peptides (42, 43). In addition to these universal inhibitors, specific microenvironmental stressors, such as hypoxia and extracellular acidity, also directly inhibit MHC-I expression and antigen presentation, thereby enabling immune evasion (44). EMT has also been reported to be associated with the inhibition of immunoproteasome function, decreasing peptide diversity, as well as conferring a poor prognosis (45). Furthermore, the advent of post-translationally modified ligands is another twist, as such changes may give rise to novel epitopes not expected from alterations at the genome level in and of themselves (46, 47). Together, these mechanisms not only enable cancers to evade immune recognition but also expand the treatment window by generating distinct epitopes beyond the conventional mutation-driven neoantigens. In this review, we incorporate new findings on how classic and non-traditional antigen processing, microenvironmental stress, and PTM work together to remodel the tumor immunopeptidome. We also emphasize how said mechanisms create both vulnerabilities and opportunities, redefining the immunopeptidome as an ever-changing interface at the nexus of precision immuno-oncology.

## 2 Integrative framework and roadmap for tumor immunopeptidome remodeling

The immunopeptidome has emerged as a central determinant of tumor–immune interactions, moving far beyond the classical proteasome–TAP–ER–MHC-I axis that once dominated models of antigen presentation. Rather than a static repertoire, recent research shows that the tumor immunopeptidome is dynamically remodeled by tumor-intrinsic alterations and microenvironmental tensions. Cytokine signaling, for instance, and pharmacologic manipulation can trigger multifactorial remodeling of peptide landscapes that regulate density and diversity of presented antigens to CD8^+^ T cells (42). Hypoxia, a characteristic of solid tumors, suppresses MHC-I surface expression and reduces antigen visibility (44), while EMT disrupts immunoproteasome stability, eliminates peptide diversity, and is associated with a poor prognosis (45). The tumor microenvironment (TME) extracellular acidity hinders the Interferon (IFN)-γ-mediated activation of immunoproteasome subunits and T-cell recognition (48).

In addition to microenvironmental stresses, the biochemical composition of the supplied ligands also triggers another heterogeneity. Post-translationally modified peptides, such as phosphorylated and glycosylated ligands, have been identified as bonafide tumor antigens with therapeutic potential (46). Large-scale immunopeptidomic research also identified numerous post-translationally spliced peptides from distinct cancer types, indicating that genomic sequence cannot predict the immunopeptidome (47). Furthermore, pharmaceutical treatments such as second mitochondria-derived activator of caspases (SMAC) mimetics (e.g., Birinapant) quantitatively and qualitatively reengineer the tumor immunopeptidome, expanding the landscape of presented epitopes and boosting tumor visibility (43).

At a systems level, population-scale resources are currently offering unprecedented resolution of the immunopeptidome. The Ligand.MHC atlas has documented hundreds of thousands of distinct peptides across various malignancies and HLA alleles, providing foundational frequencies for therapeutic development (49). The Peptides for Cancer Immunotherapy Database (PCI-DB) concurrently develops standardized primary-tissue immunopeptidomic datasets to inform next-generation peptide-based immunotherapies (50).

All in all, these results define the immunopeptidome as an indicator and biomarker of tumor visibility. In this review, we follow a structured roadmap (1): presenting classical and alternative antigen-processing pathways (2); explaining how microenvironmental stressors such as hypoxia, acidity, and EMT reshape antigen presentation (3); exploring PTMs partnered with spliced peptides as emerging antigen classes based on recent discoveries; and finally integrating cancer-type–specific landscapes with technological innovations. This perspective represents the immunopeptidome as a rewritable code, not solely a passive result of protein degradation, at the center of precision immuno-oncology.

## 3 Core mechanisms shaping the tumor immunopeptidome

The tumor immunopeptidome is shaped by both the cancer type and key molecular mechanisms that control antigen processing and presentation. Central pathways, including classical proteasome–TAP–ER–MHC-I transport, trimming by ERAP1/2, and activation of the immunoproteasome, influence the density and chemistry of epitopes that are displayed. These pathways are dynamically modified through tumor-intrinsic alterations (for example, the loss of TAP during progression), tumor microenvironment forcing (hypoxia, acidification, EMT), and stress-induced responses that can enhance or limit immune visibility. In addition, the heterogeneity of the proteasome, PTMs, and subunit-specific signatures provides biomarker value and prognostic information across cancer types. Furthermore, these elicited mechanisms are dynamic and can be therapeutically exploited: pharmacological induction or inhibition of proteasomes, subunit-selective targeting of immunoproteasomes, or context-dependent control of ERAP/TAP may be able to re-establish or reinstate antigen processing/presentation. As a guide for the reader, a summary of all these mechanisms and studies, along with their findings and translational potential, can be found in **Table 1** to provide an introduction to the discussion in each subsection.

**Table 1:** Overview of core mechanisms shaping the tumor immunopeptidome, including canonical and alternative antigen processing, immunoproteasome regulation, and translational opportunities.

Focus	Key findings	Implications	Ref.
Pharmacologic immunoproteasome activation in multiple myeloma	Activation expanded MHC-I peptide repertoire, unmasked neoantigens (>100-fold increase), boosted T-cell killing and revealed actionable targets.	Immunoproteasome activation can diversify immunopeptidome and potentiate personalized immunotherapy.	(51)
Proteasome/immunoproteasome heterogeneity in gastric cancer subtypes	Diffuse-type gastric cancer enriched for immunoproteasomes, conferring resistance to inhibition and enhanced migration; epithelial-type showed balanced proteasome activity.	Context-specific proteasome patterns may dictate resistance to therapy; dual role in immunity vs tumor survival.	(52)
mRNA vaccine with proteasome-targeting sequence	Proteasome-targeted mRNA vaccine enhanced antigen processing, increased MHC-I pathway activation, promoted strong CD8+ T cell responses, tumor suppression, and immune memory.	Targeting proteasomal routing enhances vaccine potency and may optimize cancer vaccine design.	(53)
Role of FAT10 in MHC-I antigen presentation	FAT10 deletion did not affect MHC-I surface expression or epitope diversity; antiviral CTL responses intact → FAT10 dispensable for immunopeptidome shaping.	Clarifies that not all ubiquitin-like modifiers are essential; sharpens focus on functionally relevant proteasome regulators.	(54)
β5i expression in NSCLC (J Clin Pathol, 2021)	High β5i expression in ~20% of NSCLC, enriched in adenocarcinoma, correlated with improved 5-year survival; dual inhibition enhanced cytotoxicity.	β5i expression serves as both favorable prognostic marker and potential therapeutic target in NSCLC.	(55)
Hypoxia-induced downregulation of antigen presentation	Hypoxia suppressed MHC-I surface levels and reduced immunopeptidome diversity via PERK-autophagy pathway; reversing hypoxia restored presentation.	Targeting hypoxia–autophagy axis may restore antigen visibility and improve immunotherapy response.	(44)
EMT-driven loss of immunoproteasome in NSCLC	EMT phenotype associated with loss of immunoproteasome, reduced peptide repertoire (50–60 vs 400–500 peptides), poor prognosis, and immune evasion.	Reversing EMT-driven suppression (e.g., IFN-γ, epigenetic drugs) could reinstate antigen diversity and sensitize tumors to T-cell immunity.	(45)
Extracellular acidity impairs IFN-γ induction of immunoproteasome	Acidic TME (pH 6.5) blocked IFN-γ induction of β1i/β2i through STAT3 activation and impaired STAT1; reduced MHC-I surface expression.	Targeting STAT3 or buffering acidity may re-enable immunoproteasome induction and enhance immunotherapy efficacy.	(48)
PSMB8 as biomarker in thyroid carcinoma	PSMB8 upregulation correlated with immune infiltration, checkpoint expression, and favorable prognosis in thyroid carcinoma.	PSMB8 is a robust prognostic and immune-related biomarker; potential therapeutic target in thyroid cancer.	(56)
PSMB8/9/10 signatures in muscle invasive bladder cancer	High expression of PSMB8/9/10 predicted prolonged survival and better immunotherapy responses in multi-cohort studies of MIBC.	Composite IP signature can stratify bladder cancer patients for immunotherapy selection.	(57)
PSMD2 in hepatocellular carcinoma	PSMD2 overexpression in HCC is associated with a poor prognosis, checkpoint upregulation, and is predictive of immune evasion.	Not all proteasome components are equivalent biomarkers; PSMD2 marks adverse prognosis and immune evasion.	(58)
PSMB8 expression in hepatocellular carcinoma (integrated analysis)	PSMB8 upregulated in HCC, correlated with immune checkpoints and adaptive immune compartments, with prognostic implications.	Context-specific interpretation of PSMB8 is needed in HCC, which may inform combined checkpoint and microenvironment-targeting strategies.	(59)
Immunoproteasome expression in melanoma	PSMB8/9 expression linked to improved survival and checkpoint response, outperforming TMB in melanoma; functional link to enhanced TIL recognition.	IP-high state predicts checkpoint therapy benefit in melanoma, validating IP as a functional biomarker beyond TMB.	(60)
PSMB8/9 expression in triple-negative breast cancer	Tumor-cell PSMB8/9 expression associated with superior outcomes in TNBC, implicating tumor-intrinsic IP as the prognostic compartment.	Supports the notion that IP-high tumors are responsive to immunotherapy in TNBC; encourages the tumor-intrinsic scoring of IP subunits.	(61)
Proteasome pool heterogeneity in breast cancer subtypes	Proteasome activity heterogeneity associated with molecular subtype markers in breast cancer, highlighting IP as stratification biomarker.	Proteasome heterogeneity adds a layer of complexity to biomarker stratification for breast cancer.	(62)
Pan-cancer prognostic associations of immunoproteasome expression	Pan-cancer analysis showed prognostic value of IP expression context-dependent; favorable in inflamed TMEs, adverse in suppressive/metabolic contexts.	Highlights the need for two-dimensional biomarker models that integrate IP levels and immune context for prognostication.	(63)
M3258 as selective LMP7 inhibitor in multiple myeloma	M3258 potently suppressed LMP7, induced apoptosis, durable tumor control; superior to broad PIs with lower off-target toxicity.	Demonstrates feasibility of subunit-specific PI as potent and safer therapeutic strategy in hematologic cancers.	(64)
ONX-0914 and selective immunoproteasome inhibition in prostate cancer	ONX-0914 inhibited tumor progression, reduced immunosuppressive myeloid cells, blocked IL-6/IL-23/IL-17 inflammation, and induced apoptosis.	Shows immunoproteasome inhibition can act as anti-inflammatory and antitumor therapy, extending beyond hematologic tumors.	(65)
M3258 efficacy in acute lymphoblastic leukemia	M3258 triggered proteotoxic stress and apoptosis in KMT2A::AFF1 ALL, comparable to bortezomib with improved selectivity.	Highlights M3258 as a targeted therapy for genetically defined leukemias, broadening the applications of PI.	(66)
Combination strategies with proteasome inhibitors in breast cancer	Bortezomib + TM or AMD3100 reduced proteasome activity, activated AMPK–STAT3 axis, enhanced CD8^+^ T-cell recruitment, and antigen presentation.	Indicates PI therapy can serve as immune sensitizer; rational combinations expand therapeutic benefit in solid tumors.	(67)
Proteasome inhibition overcoming resistance in B-cell malignancies	PIs restored efficacy in PI3K-inhibitor–resistant B-cell malignancies; combined Bcl-2i + PI achieved partial response in refractory CLL patient.	Validates PIs as salvage strategy for resistant B-cell malignancies; combination approaches needed for durable control.	(68)
Synergy of proteasome inhibitors with oncolytic reovirus therapy	Carfilzomib enhanced reovirus replication in monocytes, improved viral delivery to myeloma cells, potentiated immune-mediated tumor clearance.	Expands PI use as immune co-factor in virotherapy; supports combinatorial immuno-oncology strategies.	(69)
M3258 reshaping tumor microenvironment in TNBC and IBC	M3258 reduced tumor growth, decreased M2 macrophages, increased CD8^+^ infiltration, reprogrammed immune TME toward antitumor activity.	Confirms that selective IPIs reprogram the TME in solid tumors, aligning with immunomodulatory goals.	(70)
Chronic inflammation-driven tumorigenesis (CAC)	WT mice with intact IP developed tumors under chronic inflammation; IP triple-KO mice resistant.	IPs amplify pro-inflammatory mediators, recruiting innate immune cells → sustain protumor loop.	(71)
CRPC and Th17-driven inflammation	Th17-type inflammation induced LMP7; ONX-0914 inhibited IL-17 angiogenesis/EMT, reducing tumor progression.	LMP7 links inflammatory circuits to tumor progression; inhibition offers therapeutic avenue.	(72)
CRC – Epigenetic rewiring	Proteasome inhibition reduced DNMT1/3B via AKT/mTOR blockade, altering DNA methylation and transcriptomics.	Proteasome–epigenetic cross-talk sustains tumor resilience; inhibition rewires transcriptome.	(73)
CRC – Metabolic regulation	Glutamate-to-glutathione flux inhibition activated ROS and enhanced IP activity → improved antigenicity and T-cell recognition.	Targeting metabolism can heighten immunogenicity via IP activation.	(74)
Melanoma – Tumor suppressor role of PSMB9	PSMB9 upregulated via hypomethylation, enhanced CD8+ activation, IFN-γ signaling; inhibited melanoma proliferation/migration.	PSMB9 functions as IFN-γ-sensitive tumor suppressor and biomarker.	(75)
AML – PSMB10 and stemness	High PSMB10 maintained leukemia stemness, immune evasion; inactivation promoted senescence, drug uptake, restored CTL killing.	PSMB10 sustains resistant stem-like leukemia pools; inhibition therapeutic.	(76)
CRC – MCL-1 expression	MCL-1 paradoxically associated with immune features (PD-L1, MSI-H, TMB-high); inhibition restored MEKi sensitivity in TNBC/IBC.	MCL-1 is context-specific: biomarker in CRC, therapeutic target in resistant TNBC/IBC.	(77, 78)
Leukemia ICV vaccine	IP induction by oncolytic viruses dispensable for therapeutic efficacy in ICV models.	In certain vaccine contexts, IP not required for efficacy.	(79)
Multiple Myeloma – PSMB6/PSMB9 polymorphisms	GA+AA genotypes of PSMB6 (rs3169950) and PSMB9 (rs17587) linked to poor response to bortezomib-based therapy; no OS/PFS difference.	Genetic polymorphisms may predict treatment response; useful for patient stratification.	(80)
AML – UBE2N and proteostasis	UBE2N stabilizes proteins via K63 ubiquitination; its inhibition triggers K48-linked degradation through immunoproteasome, suppressing AML.	UBE2N is a vulnerability in immunoproteasome-positive AML; inhibition may serve as therapeutic strategy.	(81)
Pancreatic cancer – TGF-β2 and gemcitabine sensitivity	TGF-β2 inhibition reduced proliferation, stem-like cells, ECM remodeling; synergized with gemcitabine to reduce growth and metastasis.	Targeting TGF-β2 enhances chemosensitivity and suppresses metastasis; potential adjunct to chemotherapy.	(82)
PD-1 stability and USP24	IL-6/STAT3 induced USP24 expression stabilized PD-1 by removing K48 ubiquitin; USP24 inhibition restored CTL activity and improved immunotherapy response.	USP24 maintains T cell exhaustion; targeting it can enhance antitumor immunity and improve checkpoint therapy.	(83)

### 3.1 Classical and alternative antigen processing

For decades, the classical MHC-I antigen presentation pathway emerged as the critical orchestrator of tumor immuno-surveillance. This is a highly coordinated process whereby proteasomal processing results in the production of short peptides that are shuttled into the ER by TAP, trimmed by aminopeptidases such as ERAP1/2, and loaded onto MHC-I molecules for presentation to cytotoxic CD8^+^ T cells in a series of tightly orchestrated steps. However, this linear proteasome–TAP–ER–MHC-I process is more than an efficient conveyor belt. It is the process that dictates the density and diversity of epitopes that shape the immunopeptidome and determine whether a tumor is visible or recognizable to the immune system. Malignant cells often exploit weaknesses in this pathway to evade recognition. The TAP, an ATP-binding cassette (ABC) transporter, is essential for processing and presenting MHC class I-restricted antigens (32). TAP1 and TAP2 transporter that shuttles peptides from the cytosol into the ER for MHC-I loading (84). While TAP1 and TAP2 work together as a functional heterodimer in the MHC-I antigen-processing pathway, they have distinct biological and clinical roles. TAP1 is a more significant peptide gatekeeper for entering the ER, and the loss of TAP1 will entirely abrogate MHC-I antigen presentation, contributing to immune evasion and tumorigenesis, as demonstrated by Johnsen et al. (85). TAP2 plays a more modulatory role, affecting peptide repertoire content, immune-cell invasion, and checkpoint signaling. The following recent integrative review article by Yang et al. (86). has shown that defective TAP2 expression is associated with unfavorable prognosis and immunotherapy responsiveness in cancers. Collectively, these observations illustrate a mechanistic difference where TAP1 loss reduces antigen presentation, while TAP2 dysregulation redistributes immune landscape and therapy responsiveness.

In addition to peptide transport, the trimming step mediated by ERAP drastically reorganizes the immunopeptidome. As discussed previously, the absence of ERAP1 in humans alters the HLA-E ligandome, which eliminates the presentation of the canonical VL9 peptide, thereby disrupting the immunoinhibitory NKG2A–HLA-E checkpoint. This reorganization can make the cancer responsive to immune checkpoint blockade when CD8^+^ and Natural Killer (NK) cell antitumor activity is released (38). On the other hand, ERAP1 can perform a reverse function: by cleaving with maximal efficiency, ERAP1 induces single epitope immunodominance, overrepresenting the aggregate repertoire and distorting T cell recognition (87). This duality accounts for the context-dependent nature of ERAP activity and suggests that precisely modulated inhibition, rather than blunt inhibition, would prove to be most beneficial therapeutically. Polymorphic variations within immune system proteins contribute substantially to the heterogeneity of individual immune responses. The ERAP1 is responsible for trimming precursor peptides into suitable antigenic fragments for MHC class I presentation (88). Naturally occurring ERAP1 variants form complex allotypes characterized by multiple non-synonymous single-nucleotide polymorphisms (SNPs), collectively defined as haplotypes. Ten major ERAP1 haplotypes, labeled Hap1 through Hap10, represent more than 99% of the allelic diversity observed across human populations (89). The polymorphic residues are typically situated near the catalytic core (positions 346 and 349), within the peptide-binding groove (positions 725 and 730), or in interdomain regions that mediate conformational rearrangements crucial for enzymatic activation (positions 528 and 575). Consequently, such variations can modulate ERAP1 function through several distinct mechanisms. Multiple *in vitro* investigations have demonstrated that the K528R substitution exerts a particularly pronounced effect (90–94), primarily by altering the conformational dynamics that regulate the equilibrium between the enzyme’s active and inactive states (95). These haplotypes encode functionally distinct allotypes whose trimming efficiencies vary widely, up to ~60-fold, and depend on substrate sequence and length (88). Remarkably, allotype 10, previously associated with Behçet’s disease, consistently exhibits markedly reduced enzymatic activity, indicating that a considerable portion of the population carries a functionally inactive variant of the ERAP1 gene (88). Biochemical analyses have demonstrated that ERAP1 allotypes vary in both their catalytic efficiency and substrate-binding affinity, leading to distinct patterns of intermediate accumulation during the multistep peptide-trimming process (88). Moreover, differential responsiveness to an allosteric inhibitor acting on the regulatory domain suggests that allotypic polymorphisms modulate intramolecular communication between the regulatory and catalytic sites (88). Based on their overall enzymatic behavior, ERAP1 allotypes can be categorized into three major functional classes: “normal,” “hypo-,” and “hyper-trimming” variants (90, 96). For instance, Hap10 acts as a hypo-functional allotype with notably reduced trimming activity in Behçet’s disease contexts (97, 98).

Furthermore, ERAP polymorphisms interact with specific HLA alleles to shape the immunopeptidome in an allele-dependent manner. Structural and functional studies indicate that polymorphic variants and allotypes of ERAP1 and ERAP2 modulate peptide trimming in an HLA allele-dependent manner (41, 99, 100), thereby shaping distinct immunopeptidomes across individuals. For example, Reeves et al. (100) demonstrated that naturally occurring ERAP1 allotype pairs associated with ankylosing spondylitis display reduced catalytic efficiency and are less capable of generating optimal-length peptide ligands for the AS-linked HLA-B*27:05 molecule compared with allotypes enriched in healthy controls (100). These findings provide direct functional evidence that polymorphic ERAP1 combinations influence peptide processing in an HLA allele-specific manner, thereby shaping the antigenic repertoire presented to cytotoxic T cells (100). Immunopeptidomic analyses further confirm that variation in ERAP1/2 expression or activity causes distinct, HLA-specific alterations in peptide repertoires (101, 102). Moreover, ERAP2 has been shown to increase the abundance of a peptide sub-motif selectively presented by HLA-A29, providing direct evidence of allele-restricted peptide editing (103). Collectively, these findings highlight a coordinated, co-evolutionary relationship between ERAP allotypes and HLA class I molecules that governs antigenic visibility and contributes to inter-individual variation in immune recognition.

Beyond the roles of TAP and ERAP, the efficient loading of peptides onto MHC class I molecules fundamentally relies on the peptide-loading complex (PLC). This transient, multiprotein assembly, located within the ER, plays a pivotal role in orchestrating a hierarchically organized immune response (104). The PLC is composed of the peptide transporter heterodimer TAP1/2, the protein disulfide isomerase ERp57 (also referred to as PDIA3), the lectin chaperone calreticulin (CALR), and the MHC-I–specific cofactors tapasin [also known as TAP-binding protein (TAPBP)] and TAP-binding protein–related (TAPBPR) (105–107). Initial biochemical and functional investigations into tapasin, particularly its covalent disulfide linkage with ERp57 (108), were later corroborated by structural studies elucidating the three-dimensional conformation of the tapasin–ERp57 heterodimer (109). These analyses revealed that ERp57 provides crucial structural stabilization for tapasin. Complementary mutagenesis experiments further delineated specific tapasin regions required for restoring MHC-I surface expression in tapasin-deficient cells (110). Additionally, the ER-resident chaperones calnexin (CNX) and CALR facilitate the folding and assembly of most Asn-linked glycoproteins during their maturation within the ER (111). Together, these chaperones define a quality-control checkpoint that favors high-affinity peptide selection and stable surface expression of MHC-I. Disruption of any PLC component, such as TAPBP or CALR loss, frequently observed in tumors, diminishes peptide diversity and promotes immune evasion (112, 113). Incorporating the PLC into models of immunopeptidome regulation thus provides a more complete view of how antigen processing fidelity is ensured and how its dysregulation contributes to tumor invisibility.

While canonical processing dominates most settings, accumulating evidence reveals alternative mechanisms that can sustain immune recognition in its absence. CD8^+^ memory T cells can recognize and destroy tumor cells with no TAP or beta-2 microglobulin (β2M) at all, driven by Sec62-mediated import of peptides into the ER and chaperone-facilitated stabilization of free MHC-I heavy chains. This degree of presentation, although suboptimal, is sufficient to induce cytotoxicity both *in vitro* and in tumor-bearing mice (114). Parallel research in rhesus macaques identified the Mamu-B*098 allomorph, which is involved in the TAP-independent presentation of N-myristoylated lipopeptides and the activation of cytotoxic T cells under conditions of TAP deficiency (115). These results contradict the conventional binary antigen presentation model, which posits intact or deficient systems, and instead demonstrate an adaptable and robust system that retains partial immune recognition through unconventional mechanisms.

Adding further complexity, cancer cells frequently display post-translationally modified peptides through the MHC-I pathway. Large-scale immunopeptidomics sweeps have identified thousands of post-translationally modified peptides, including phosphopeptides, O-GlcNAcylation, methylation, and kynurenination, a large percentage of which are common among tumors and patients (46). These tumorigenic peptides arise directly from dysfunctional oncogenic signaling and metabolism, making them attractive targets for immunotherapy. The characterization of PTM peptides as repeated, tumor-specific ligands designates tumor immunopeptidomics not simply as a quantitative spectrum dictated by TAP or ERAPs, but as a qualitative landscape dictated by the biochemical changes of cancer.

**Figure 1:**
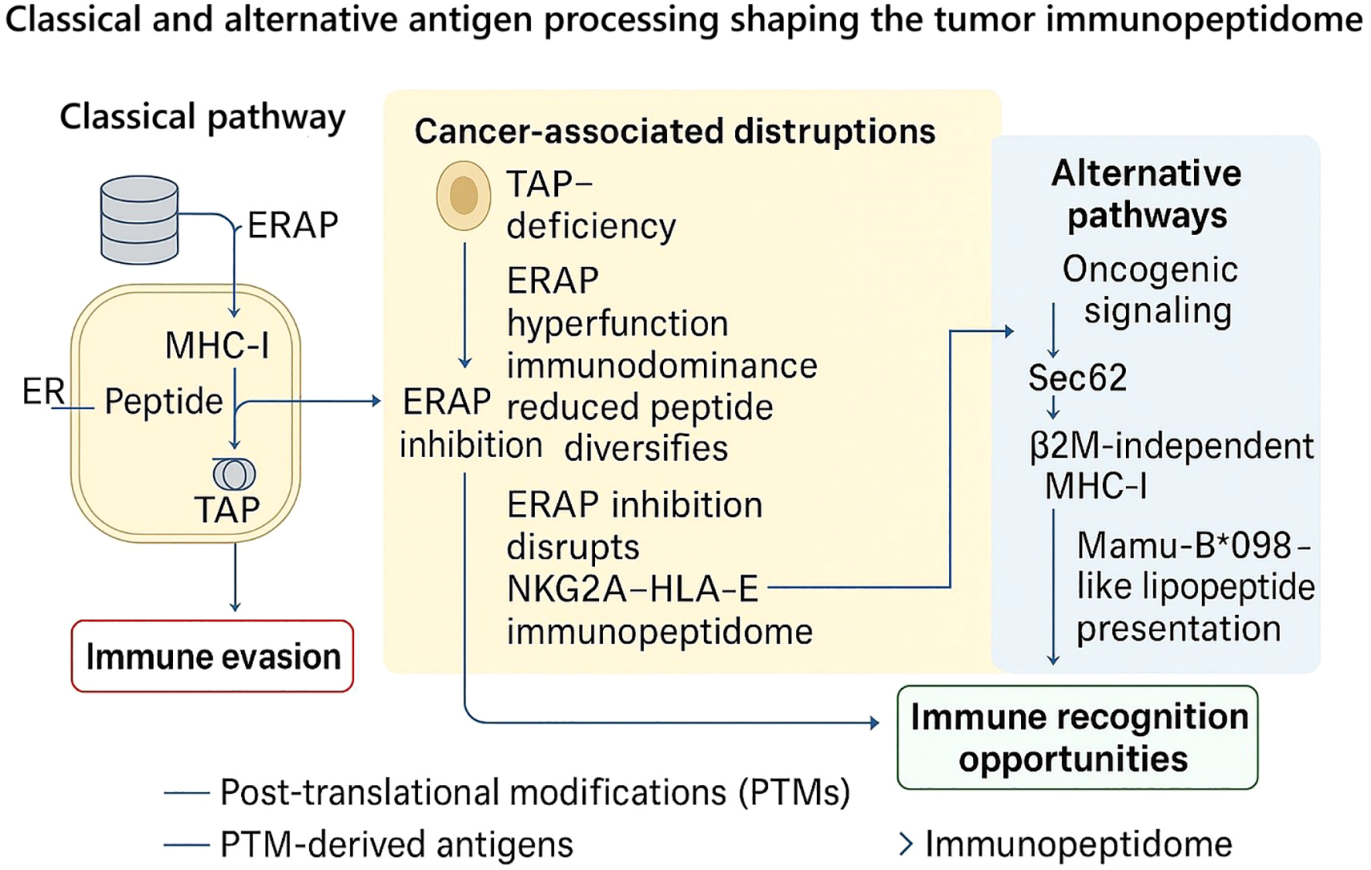
Classical and alternative antigen processing pathways shaping the tumor immunopeptidome. In the canonical pathway (left), peptides generated by the proteasome are transported into the endoplasmic reticulum (ER) via TAP and further trimmed by ERAP1/2 before loading onto MHC-I molecules for surface presentation. Tumor cells frequently disrupt this cascade (center): loss of TAP expression reduces peptide import and MHC-I stability, while ERAP1 hyperfunction drives immunodominance and reduced peptide diversity. Conversely, ERAP1 inhibition can diversify the peptide repertoire and abolish the NKG2A–HLA-E checkpoint, thereby restoring immune recognition. Alternative pathways (right) highlight the plasticity of antigen presentation in cancer: oncogenic signaling generates post-translationally modified (PTM) peptides; Sec62-dependent transport sustains peptide entry into the ER even in TAP-deficient settings; β2M-independent heavy chain binding enables residual recognition; and Mamu-B*098-like allomorphs mediate TAP-independent lipopeptide presentation. Collectively, these mechanisms illustrate how tumors both evade and expose themselves to immune detection, positioning ERAP1/2 modulation, TAP2-focused targeting, and PTM epitope exploitation as promising therapeutic strategies.

Overall, these studies characterize the tumor immunopeptidome as an active, flexible interface, rather than as a passive output of classical antigen processing. Loss of TAP function does eliminate classical presentation, but promotes clones that evade immunity (85, 86); ERAP1 function during processing can increase or limit the weaponry of and repertoire of the immune cell (38, 87); and alternative pathways can deploy Sec62 or other, non-peptidic substrates to expand the capacity for recognition in the absence of functional TAP or β2M (114, 115). Post-translationally-modified variants of substrates further expand the chemical lexicon of MHC-I ligands (46). Collectively, the evidence suggests tumors do not just “shut down” antigen presentation, but they rewire this spatial interface, producing new vulnerabilities for strategic targeting. Targeting ERAP function, exploiting maximal TAP2-based biomarkers, and investigating epitope mapping efforts of PTM-ligands are all potential translational approaches for reinstating, or “reprogramming,” tumor visibility to cytotoxic lymphocytes. Viewed from this perspective, the tumor-immunopeptidome represents both the mechanism of immune escape and the substrate for therapeutic targeting. These relationships between classical processing, cancer-associated disruptions, and alternative routes are summarized in **Figure 1**.

### 3.2 Proteasome and immunoproteasome contributions

#### 3.2.1 Functional roles of proteasome *vs* immunoproteasome in shaping the immunopeptidome

Antigen processing by the ubiquitin–proteasome system is an active determinant of MHC-I peptide presentation; the balance between constitutive proteasomes and immunoproteasomes reprograms both the quantity and quality of peptides that reach the cell surface. Immunoproteasomes induced by inflammatory cues and defined by catalytic subunit substitutions favor hydrophobic C-terminal residues that load efficiently into MHC-I, thereby adjusting epitope hierarchies and the breadth of immune visibility. Pharmacologic activation of this machinery has now shown that “turning up” immunoproteasome function can diversify the tumor immunopeptidome and expose antigens that were previously below the threshold of detection. In multiple myeloma, selective activation enhanced (i) the number and diversity of MHC-I ligands; (ii) unveiled neoantigens greater than >100-fold at an individual loci; and (iii) enhanced T-cell cytotoxicity in cell lines, primary patient samples, and xenografts, all establishing immunoproteasome stimulation as a means to expand immune surveillance, and facilitate personalized targeting (51).

Simultaneously, the tumor lineage and proteasome architecture create contextual restrictions on how this axis shapes peptide landscapes. In gastric cancer model systems, diffuse-type cells selectively formed immunoproteasome subunits into 19S-capped particles in a unique manner, switching active proteasome forms analogous to 26S/30S and with associated increased resistance to proteasome inhibitors and increased migratory state; while epithelial-type cells had a more evenly-balanced 19S/11S capping profile that gave differential drug sensitivities (52). These data indicate that immunoproteasome enrichment can be immunogenic in one context (via repertoire expansion) yet pro-survival in another (via proteostasis advantages), underscoring that the same biochemical switch can remodel the immunopeptidome toward opposite clinical ends depending on cellular state.

Deliberate routing of vaccine antigens into proteasomal degradation provides a complementary way to exploit this biology. An mRNA platform that fuses antigens to a proteasome-targeting peptide enhanced antigen destruction through the ubiquitin–proteasome system, boosted genes in the MHC-I pathway, and generated stronger CD8^+^ T-cell responses *in vivo*, with increased dendritic/macrophage activation, intratumoral T-cell infiltration, tumor growth delay, and durable memory (53). By engineering the entry point of the pathway, these vaccines reshape the density and kinetics of presented peptides, thereby altering the amplitude of antitumor immunity.

Finally, not every presumed contributor to proteasomal routing materially affects the immunopeptidome. Using human and mouse FAT10-deficient systems, loss of the ubiquitin-like modifier FAT10 had no detectable impact on MHC-I surface abundance, recovery after peptide starvation, or the presentation of endogenous/viral epitopes; virus-specific Cytotoxic T lymphocyte (CTL) responses *in vivo* were preserved, arguing that FAT10’s role in MHC-I antigen generation has been overestimated (54). Negative results of this kind refine mechanistic priors and focus therapeutic efforts on nodes with demonstrable control over peptide output.

**Figure 2:**
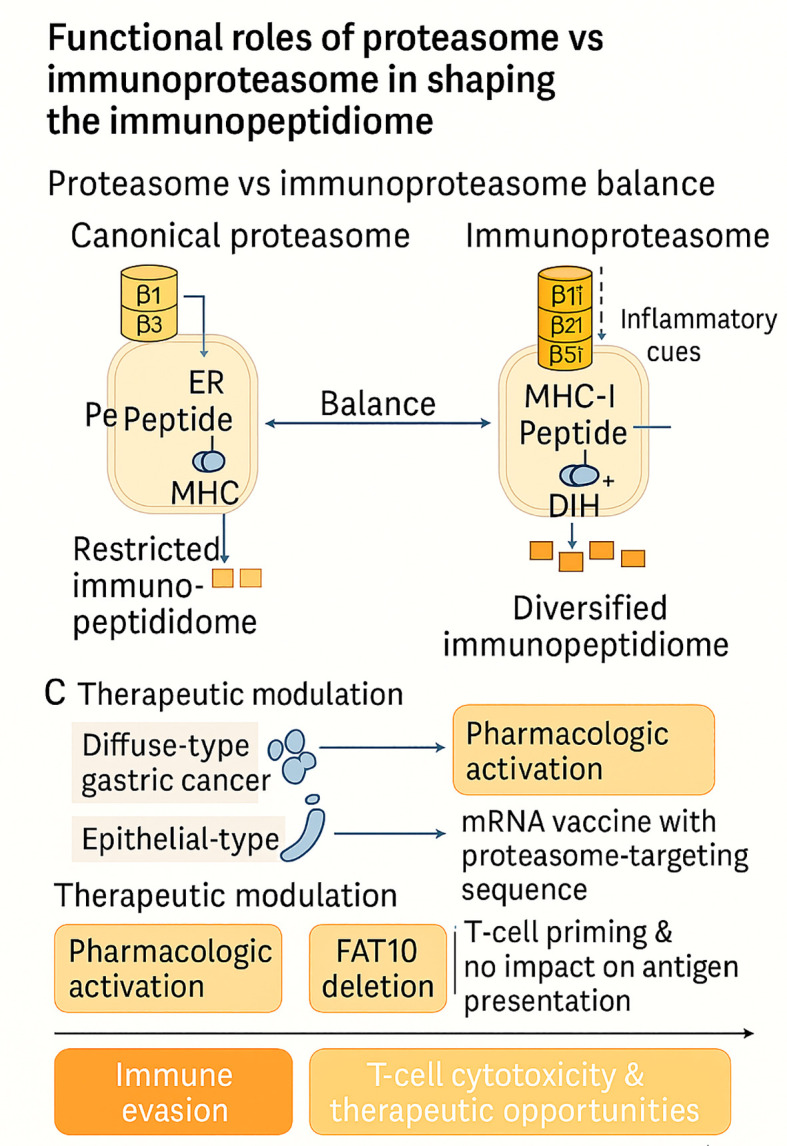
Functional interplay between constitutive proteasomes and immunoproteasomes shapes the tumor immunopeptidome. Inflammatory cues induce immunoproteasome subunits (β1i, β2i, β5i), diversifying antigen presentation, whereas lineage-specific architectures (e.g., diffuse-type gastric cancer) and therapeutic interventions (pharmacologic activation, mRNA vaccines, FAT10 deletion) variably reprogram peptide repertoires. Together, these mechanisms dictate immune evasion versus enhanced T-cell cytotoxicity and therapeutic opportunities.

Taken together, these studies position the proteasome–immunoproteasome axis as a programmable determinant of tumor immunogenicity. Activation of immunoproteasomes can expand and re-rank peptide repertoires to reveal neoantigens and enhance T-cell efficacy (51), while drug-type–dependent assembly states can shift the balance in a drug-resistant manner, regardless of immunogenicity (52). The development of vaccines that can bias the antigen into proteasomal channels demonstrates translational tractability (53); conversely, the dispensability of FAT10 clarifies which modifiers are unlikely to alter the immunopeptidome (54). The overall implication is that truly successful manipulation of this axis will require measures that consider context, including lineage, proteasome capping, and inflammatory tone, when targeting antigen activation, inhibition, or retargeting. These functional contrasts and therapeutic opportunities across the proteasome–immunoproteasome axis are schematically summarized in **Figure 2**.

#### 3.2.2 Tumor-driven modulation and immune evasion

The TME has a significant impact on the proteasome-immunoproteasome pathway, altering the antigenic landscape to promote immune evasion. These changes, rather than being passive abnormalities, are adaptive tactics employed by cancer cells to evade cytotoxic T-cell recognition by downregulating antigen processing and presentation. Hypoxia, a common feature of solid tumors, is one well-studied driver. New immunopeptidomic investigations reveal that oxygen deprivation suppresses MHC-I surface appearance and reduces the variety of presented peptides via pancreatic EIF2-α kinase (PERK)-dependent autophagic activation (44). This inhibits CD8^+^ T-cell identification, whereas restoring mitochondrial respiration by medication improves oxygenation and antigen presentation. These results establish a molecular relationship between hypoxia and immune evasion, highlighting the potential for targeting hypoxia-autophagy pathways to restore tumor visibility.

An alternative pathway involves EMT, which disrupts the expression of immunoproteasomes in non-small cell lung cancer (NSCLC). Proteomic comparisons of 42 cell lines showed that mesenchymal-like cells had significantly lower activity of the immunoproteasome, with a limited capacity to generate 50–60 unique MHC-I-bound peptides compared to 400–500 peptides generated by epithelial cells (45). Loss of the immunoproteasome subunit correlated with recurrence, metastasis, and decreased survival in patients, placing EMT-mediated repression on a double pedestal as a dual mediator of invasion and immune invisibility. Notably, the restoration of immunoproteasome expression by IFN-γ and epigenetic controllers re-established antigen diversity and re-sensitized mesenchymal cells for CD8^+^ T-cell killing, offering translational pathways for preventing this immune evasion mechanism (45).

In addition to lineage plasticity, extracellular acidity in the TME presents another barrier. At pH 6.5, several tumor models demonstrated aberrant IFN-γ induction of immunoproteasome subunits β1i and β2i, accompanied by diminished STAT1 activation and perturbed STAT3 signaling (48). This transcriptional downregulation resulted in lowered surface MHC-I expression, thereby impairing CD8^+^ T-cell priming. Such findings suggest that acidity not just dysregulates effector T-cell function outright but also contaminates the antigen-presenting machinery in its primary configuration. Manipulation of STAT3 activity or buffering of TME acidity might thus restore IFN-γ sensitivity and the efficacy of immunotherapy in acidified tumors (48).

Expression of some of these immunoproteasome subunits alone could be prognostic. In NSCLC, β5i was overexpressed in ~20% of cases and was more prevalent in adenocarcinoma (40%). It was also associated with better five-year survival in earlier-stage disease (55). β5i-high tumors were also more responsive to dual inhibition with ONX0914 and MG132 *in vitro*, indicating that immunoproteasome expression is both a valuable biomarker and a targetable weakness. These results characterize the paradoxical function of immunoproteasome modulation: its absence in mesenchymal tumors leads to immune evasion, whereas its presence in adenocarcinoma increases antitumor immunity and drug responsiveness (55).

**Figure 3:**
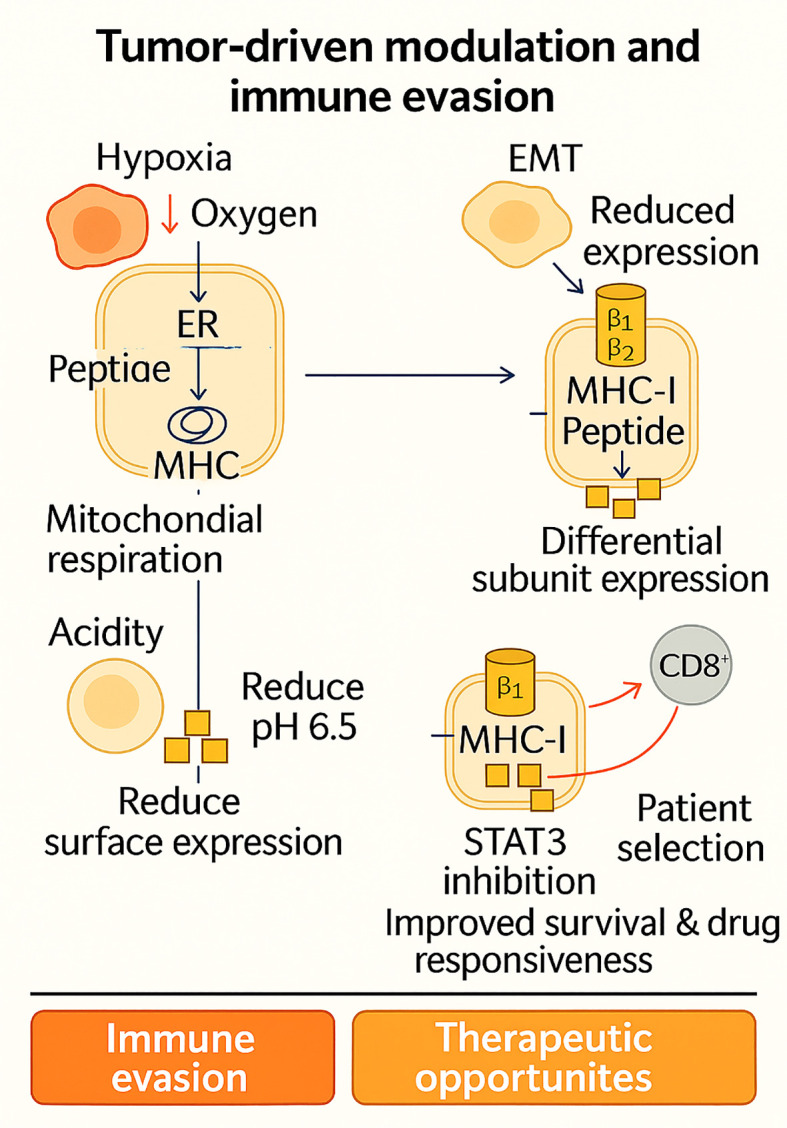
Tumor-driven modulation of antigen processing illustrates how hypoxia, EMT, acidity, and subunit-specific changes in the tumor microenvironment converge to downregulate MHC-I presentation and promote immune evasion. Yet, each of these adaptive strategies simultaneously reveals therapeutic vulnerabilities ranging from hypoxia-targeting and IFN-γ/epigenetic restoration to STAT3 inhibition and β5i-based patient stratification highlighting the immunoproteasome as both a barrier and an opportunity in cancer immunotherapy.

All in all, hypoxia, EMT, acidity, and subunit-specific control demonstrate dynamic immunoproteasome remodeling by the TME to facilitate immune evasion. **Figure 3** schematically depicts the mechanisms and their therapeutic implications, in which hypoxia, EMT, acidity, and subunit-specific modulation converge to prevent MHC-I presentation and facilitate immune evasion, as well as reveal lines of therapeutic intervention. Every process, however, also offers therapeutic potential: hypoxia-toxic drugs to enhance antigen visibility, IFN-γ or demethylating agents to rescue EMT-mediated loss, STAT3 inhibition in acidic tumors, and β5i-patient selection in NSCLC. Combined, these observations redirect the immunoproteasome from a latent antigen-processing machine into an active sensor of microenvironmental stress and a crucial regulator of whether tumors are immune-resistant or immune-vulnerable.

#### 3.2.3 Immunoproteasome as a biomarker and prognostic indicator

**Figure 4:**
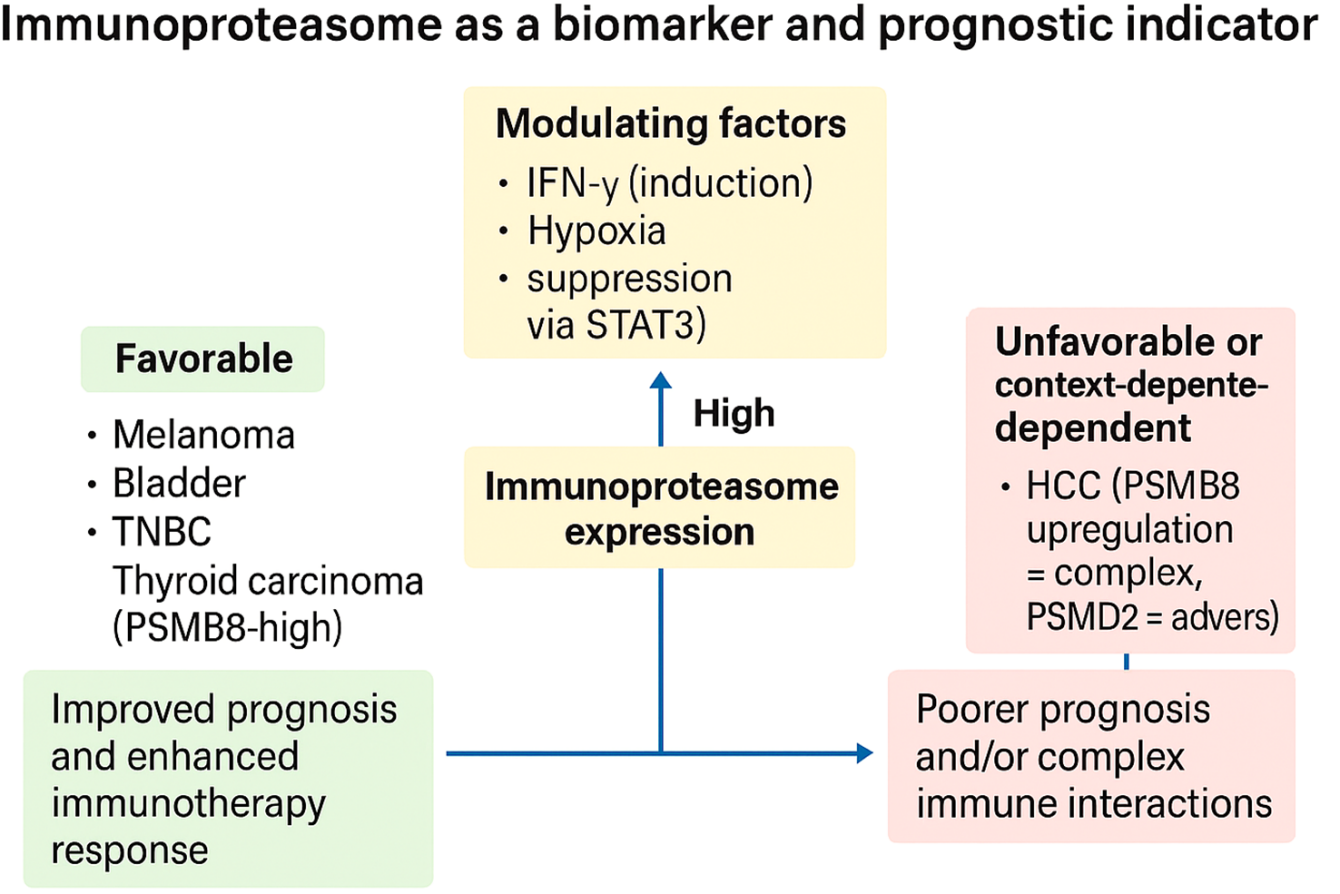
Immunoproteasome expression as a context-dependent biomarker of prognosis and immunotherapy response. High immunoproteasome (IP) expression can stratify tumors into favorable or unfavorable prognostic categories depending on tumor type and microenvironmental context. In melanoma, bladder cancer, triple-negative breast cancer (TNBC), and thyroid carcinoma, IP-high states associate with improved prognosis and enhanced response to immune checkpoint blockade. In contrast, in hepatocellular carcinoma (HCC)—particularly with PSMD2 upregulation—and across pan-cancer settings dominated by suppressive tumor microenvironments, IP expression may coincide with poorer outcomes or complex immune interactions. Modulating factors such as IFN-γ (inductive), hypoxia (suppressive), and extracellular acidity (suppressive via STAT3) dynamically influence IP activity and thereby its predictive value. Collectively, these findings underscore the immunoproteasome as both a biomarker and a barometer of immune readiness, with therapeutic implications for patient stratification and combination strategies.

Across solid tumors, immunoproteasome (IP) expression is tightly coupled to the state of the tumor–immune ecosystem, yielding context-dependent associations with outcome and therapy response. Multi-omic and clinical-inference studies converge on IP catalytic subunits (notably Proteasome subunit beta type-8 (PSMB8)/β5i, PSMB9/β1i, PSMB10/β2i) as readouts of an “IFN-γ–licensed” antigen-processing program that broadens the MHC-I peptidome and favors T-cell surveillance. In thyroid carcinoma, PSMB8 is upregulated and independently prognostic; its elevation correlates with nodal metastasis and extrathyroidal extension yet paradoxically aligns with a favorable prognosis and an inflamed microenvironment, including higher immune infiltration and checkpoint expression, features consistent with an antigen-presenting, therapy-amenable state (56). In thyroid carcinoma, PSMB8 is significantly upregulated, and its higher expression correlates with lymph node metastasis, extrathyroidal extension, increased immune infiltration/checkpoint expression, and a favorable prognosis (56). PSMB8 encodes the catalytic β5i subunit of the immunoproteasome (116), whose expression is induced by inflammatory cues such as IFN-γ and is a core determinant of MHC-I peptide processing (117). Within the thymus, epithelial and hematopoietic compartments express distinct proteasome programs: medullary/cortical thymic epithelial cells (TECs) and thymocytes utilize immunoproteasomes containing PSMB8/9/10 to shape the class-I peptidome (118), whereas cortical TECs uniquely express the thymoproteasome defined by β5t/PSMB11 (119–121). The thymoproteasome (β5t/PSMB11) is essential for positive selection of CD8 T cells and imprints a distinctive peptide repertoire during T-cell development (122, 123). Collectively, situating thyroid carcinoma-associated PSMB8 upregulation within this thymic proteasome context clarifies that PSMB8 marks an IFN-licensed antigen-processing program linked to T-cell surveillance, consistent with its favorable prognostic association observed in thyroid carcinoma (56), and aligns with broader data that immunoproteasome-high tumors often display inflamed microenvironments and improved outcomes under immunotherapy. A similar predictive signal emerges in muscle-invasive bladder cancer (MIBC): patients with high PSMB8/9/10 expression, especially PSMB9, exhibit prolonged survival and significantly better responses to immunotherapy across TCGA, hospital, and IMvigor210 cohorts, with IP upregulation tracking T-cell activation and cytotoxicity markers (57). In melanoma with intermediate clinical checkpoint sensitivity, PSMB8/9 are superior to tumor mutational burden as predictors of response; immunopeptidomics demonstrates that forced IP expression redefines the antigenic landscape and enhances patient-matched TIL recognition, mechanistically connecting IP levels to improved survival and checkpoint activity (60). Parallel pathology-scale evidence in breast cancer, including a 2,070-patient series, shows that tumor-cell (not stromal) expression of PSMB8/9 associates with superior outcomes, most notably in triple-negative disease, again implicating tumor-intrinsic IP as the relevant prognostic compartment (61). These favorable and context-dependent associations of immunoproteasome expression across tumor types are schematically summarized in **Figure 4**, which highlights both prognostic benefit (melanoma, bladder, TNBC, thyroid) and adverse or nuanced outcomes (e.g., Hepatocellular carcinoma (HCC) with PSMD2 (Proteasome 26S Subunit Ubiquitin Receptor, Non-ATPase 2) upregulation), as well as key modulators such as IFN-γ, hypoxia, and STAT3 signaling.

These correlations, however, are tumor-type specific and conditional on the immune context. A pan-cancer transcriptomic study reveals that “IP-high” tumors are dominated by cytotoxic infiltration and IFN-γ/Tumor necrosis factor alpha (TNF-α) pathway activation, but survival correlation is inverted in some entities (e.g., glioma, renal), where pro-tumor inflammatory programs or suppressive infiltrates predominate; the study reasons that local immediate immune context determines whether IP expression is beneficial or detrimental and needs biomarker models that collectively account for IP levels and immune infiltrate composition/polarization (63). HCC demonstrates this subtlety: whereas PSMB8 is induced and prognostically relevant, with single-cell measurements confining expression to adaptive immune compartments and tissue confirmation supporting an increase in tumor, its association with immune checkpoints (e.g., Programmed death-ligand 1 (PD-L1)) suggests concomitant engagement of immune-evasion circuits such that simplistic “high-is-good” heuristics are challenging (59). Moreover, the 19S regulatory subunit PSMD2 is overexpressed in HCC with poor prognosis, high checkpoint expression, and TIDE (Tumor Immune Dysfunction and Exclusion) immune evasion, indicating that proteasome-axis biomarkers are not all equal antigenicity surrogates; some of them are proteins that serve as markers of immunotherapy resistance and proteostasis adaptation (58).

Microenvironmental stressors modulate these biomarker relationships at their mechanistic root. Extracellular acidity (pH ~6.5)—a hallmark of hypoxic, glycolytic tumors impairs IFN-γ–driven induction of β1i/PSMB9 and β2i/PSMB10 by dampening STAT1 activation and engaging STAT3, thereby reducing MHC-I surface levels; under acidic conditions, even an “IP-high genotype” may function as “IP-low phenotype,” explaining immune-excluded, checkpoint-refractory tumors with seemingly intact antigen-presentation genes (48). Together with earlier data on hypoxia–PERK–autophagy crosstalk in antigen presentation, these findings suggest that physiologic repression can mask molecular IP signatures, and that correcting pH or oxygenation/oxygenation or blocking STAT3, may “unmask” IP-linked benefit signals in biomarker-stratified trials (48).

The composite picture is therefore comparative and conditional. In inflamed tumors, such as melanoma, MIBC, subsets of breast and lung adenocarcinoma, IP-high often correlates with improved survival and immunotherapy benefits, supported by functional evidence that IP broadens the immunopeptidome and enhances T-cell recognition (57, 60, 61). In contrast, in settings dominated by immunosuppression, metabolic stress, or alternative proteostasis programs (e.g., HCC with PSMD2 upregulation), proteasome-axis readouts may herald immune evasion and worse outcomes (58, 59, 63). These contrasts motivate a two-dimensional biomarker strategy: (i) a tumor-cell IP score (e.g., PSMB8/9/10 protein by immunohistochemistry (IHC) or RNA composite), and (ii) an immune-context score (cytotoxic infiltration, IFN-γ signatures, myeloid polarization, acidity/hypoxia proxies). Clinically, IP-high/immune-inflamed tumors prioritize checkpoint blockade ± vaccines; IP-high/acidic or hypoxic tumors may require microenvironmental normalization (buffering, anti-STAT3, hypoxia-targeting) to realize IP-linked susceptibility. IP-low/mesenchymal states may benefit from IP induction (IFN-γ, epigenetic priming) before, or in combination with, immunotherapy. In short, the immunoproteasome functions as a biomarker of a TME’s readiness for immune attack; its prognostic/predictive value peaks when interpreted through the lens of local immune ecology and metabolic stress.

#### 3.2.4 Therapeutic targeting of the proteasome/immunoproteasome axis

Targeting the proteasome has become a cornerstone in hematologic oncology, yet the clinical activity of conventional proteasome inhibitors (PIs) is tempered by systemic toxicity and limited efficacy in solid tumors. Recent efforts have focused on developing selective immunoproteasome inhibitors (IPIs), combination regimens to improve PI efficacy, and utilizing modulation of the proteasome for reengineering of the TME. Collectively, these studies suggest that the proteasome–immunoproteasome axis is not simply a cytotoxic target but a therapeutic gateway to reprogramming tumor–immune interactions.

The Low-molecular mass protein-7 (LMP7)-specific inhibitor M3258 exemplifies this strategy. In multiple myeloma (MM), M3258 demonstrated potent, selective suppression of LMP7 activity, leading to durable inhibition of ubiquitinated protein turnover, apoptosis, and superior tumor control compared to broad-spectrum PIs such as bortezomib (64). Subsequent studies in KMT2A, AFF1-mature acute lymphoblastic leukemia (ALL) confirmed that M3258 induces proteotoxic stress and apoptosis, similar to bortezomib, but possibly with reduced off-target toxicities (66). Extending to solid cancers, M3258 inhibited tumor growth and reprogrammed the immune microenvironment of triple-negative and inflammatory breast cancer (TNBC/IBC), where it suppressed M2 macrophage growth, enhanced CD8^+^ T-cell infiltration, and reduced pro-inflammatory signatures (70). These results indicate that selective IPIs not only cause tumor cell killing but also reprogram the TME to an antitumor phenotype.

In prostate cancer, another LMP7-targeting inhibitor, ONX-0914, potently inhibited tumor growth and metastasis in both castration-sensitive and castration-resistant models. Mechanistically, its action is not only due to direct apoptosis induction but also due to immunosuppressive myeloid cell depletion and inhibition of interleukin 6 (IL-6)/IL-23/IL-17-mediated pro-tumor inflammation (65). Cumulatively, these results suggest that immunoproteasome inhibition is an antitumorigenic and anti-inflammatory agent of prognostic value, extending beyond hematologic neoplasia.

Combination strategies are also revealing the immunomodulatory potential of PIs. In models of breast cancer, bortezomib plus tetrathiomolybdate (TM) or AMD3100 activated the 5′-adenosine monophosphate (AMP)-activated protein kinase (AMPK)–STAT3 signaling pathway, suppressing proteasome function and synergistically enhancing the recruitment of CD8^+^ T cells. Notably, the combination enhanced antigen presentation and C-C Motif Chemokine Ligand 5 (CCL5) secretion, demonstrating that treatment with a PI can be repurposed as both a cytotoxic stressor and immune sensitizer in solid tumors (67). Similarly, in B-cell malignancies resistant to phosphoinositide 3-kinases (PI3Ks) inhibition, proteasome inhibitors re-established treatment efficacy by inducing Bim/Mcl-1 modulation and remained effective across resistant subtypes. A refractory chronic lymphocytic leukemia (CLL) patient enrolled in a clinical trial initially responded to combined B-cell lymphoma 2 inhibitor (Bcl-2i) plus PI therapy, highlighting both the potential and the challenges of achieving sustained responses in resistant disease (68). Beyond conventional drugs, PIs have also been found to be synergistic with oncolytic virotherapy. In myeloma, carfilzomib increased reovirus replication in monocytes and improved viral delivery to myeloma cells. It was not cytotoxic, but rather enhanced immune-mediated killing of tumor cells, causing clinical responses in heavily pretreated patients (69). This broadens the therapeutic application of PIs as immune co-factors in combination with oncolytic viruses and, in fact, other immunotherapies.

Together, these studies paint a picture of a treatment scenario in which the proteasome–immunoproteasome axis serves a dual purpose: as a direct route of weakness in proteotoxic cancers and as a potent means of immunomodulation. Selective IPIs (M3258, ONX-0914) highlight the merit of targeting subunits to avoid systemic toxicity and reengineer the TME, and rational combinations with PIs illustrate how proteasome stress can complement immune or targeted therapies. But there are constraints: resistance mechanisms (e.g., adaptive Bcl-2 family signaling), context-dependent immune suppression, and response persistence demand stringent patient selection and treatment timing. Therapeutic targeting of the axis ultimately shifts from proteasome inhibition, which leads to brutal cytotoxicity, to proteasome modulation, which enables refined immuno-oncology. This is schematically depicted in **Figure 4**, where it is evident how proteasome inhibition directly induces a tumoricidal effect and, at the same time, utilizes the reused tumor microenvironment to decrease immunosuppression, increase antigen presentation, and augment CD8^+^ T-cell responses.

#### 3.2.5 Dual roles: pro-tumor *vs* anti-tumor

The IP illustrates a paradox in cancer biology: depending on the inflammatory and tissue context, it can either promote tumorigenesis or support immune-mediated clearance. This duality reflects the capacity of the IP to integrate signals from cytokines, metabolic stress, and epigenetic states, thereby shaping both the tumor cell proteome and the tumor–immune dialogue. On the pro-tumorigenic side, IP activity can exacerbate chronic inflammation, skew cytokine production, and sustain immune environments that favor cancer development. In colitis-associated carcinogenesis (CAC) models, wild-type mice with intact IPs developed tumors as a result of chronic inflammation, yet LMP2/MECL-1/LMP7 triple-deficient mice remained resistant (71). Mechanistically, IPs amplified pro-inflammatory mediators and promoted recruitment of innate immune cells, thereby fueling a protumorigenic loop. Likewise, in castration-resistant prostate cancer (CRPC), Th17-type tumor-induced inflammation promotes LMP7 expression; pharmacological inhibition of LMP7 by ONX-0914 suppresses IL-17-mediated angiogenesis and EMT, thereby suppressing tumor growth (72). These studies demonstrate that IP expression, particularly under chronic inflammatory cues, can sustain protumor circuits. The remaining protumor regulatory levels include genetic polymorphisms, regulation of proteostasis, and TGF-β signaling. In myeloma, shared SNPs in PSMB6 and PSMB9 were associated with a decreased response to bortezomib therapy, without affecting survival, demonstrating how germline variation can influence IP-related therapeutic effects (80). In acute myeloid leukemia, the ubiquitin-conjugating enzyme E2 N (UBE2N) stabilizes K63-linked chain proteins to safeguard them against immunoproteasome-dependent degradation; inhibition of this enzyme preferentially down-regulates immunoproteasome-positive acute myeloid leukemia (AML) and demonstrates the capacity of the ubiquitin–IP axis in maintaining leukemic proteostasis (81). Likewise, in pancreatic cancer, transforming growth factor-beta 2 (TGF-β2) plays a crucial role in maintaining stemness, chemoresistance, and metastasis. Antisense inhibition of TGF-β2 was found to be synergistic with gemcitabine in preventing tumor growth and dissemination, illustrating how immunoproteasome-associated signaling converges with growth factor networks (82).

Other layers are epigenetic and metabolic rewiring: in colorectal cancer (CRC), proteasome inhibition of DNA methytransferase (DNMT)1/3B synthesis by protein kinase B (AKT)/mammalian target of rapamycin (mTOR) blockade that remodeled DNA methylation and transcriptomic programs to connect proteasome activity with epigenetic plasticity (73); conversely, inhibition of glutamate-to-glutathione flux in CRC activated reactive oxygen species (ROS) pathways and promoted immunoproteasome activity, enhancing antigenicity and T-cell recognition (74). Together, these data suggest that proteasome–IP cross-talk can reinforce tumor resilience or, under specific perturbations, heighten tumor immunogenicity.

**Figure 5:**
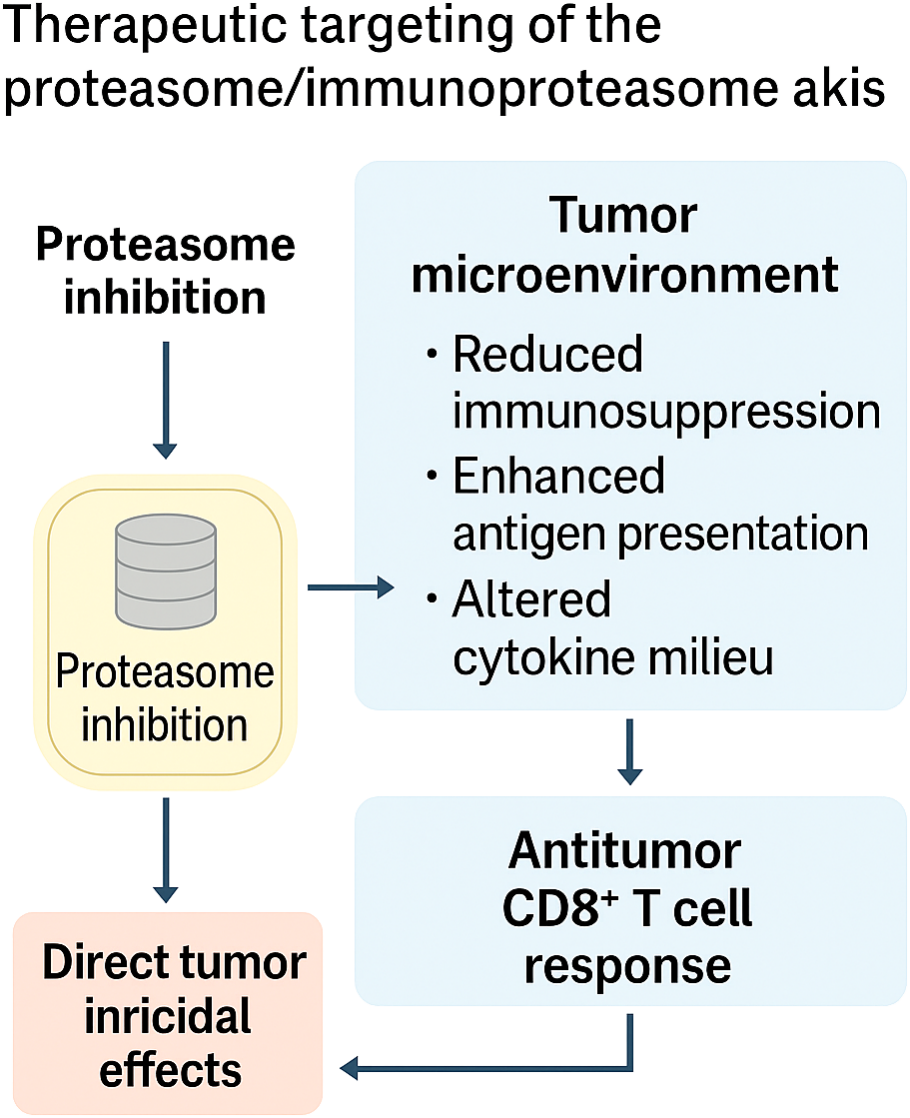
Therapeutic targeting of the proteasome/immunoproteasome axis. Selective inhibition of the proteasome or immunoproteasome exerts dual effects: (i) direct tumoricidal activity through induction of proteotoxic stress and apoptosis, and (ii) reshaping of the tumor microenvironment, characterized by reduced immunosuppression, enhanced antigen presentation, and reprogrammed cytokine milieu. Together, these effects amplify CD8^+^ T cell–mediated antitumor immunity, positioning the proteasome–immunoproteasome axis as both a cytotoxic target and an immunomodulatory lever in cancer therapy.

Conversely, increasing evidence has shown IP subunits to possess antitumorigenic activities. IP overexpression was associated with a favorable prognosis and increased CD8^+^ and Th1 immunity in melanoma, whereas it was associated with impaired T-cell cytotoxicity and APC function in IP-deficient mice (71). A finer image is drawn by multi-omics PSMB9 melanoma research, where it was recognized as an IFN-γ-sensitive tumor suppressor. Hypomethylation-induced PSMB9 upregulation enhanced CD8^+^ activation, boosted IFN-γ signaling, and suppressed melanoma cell proliferation and migration (75). Similarly, AML research has shown that high PSMB10 maintains leukemia stemness and immune evasion, but inactivation triggers senescence, drug internalization, and reactivates CTL-mediated killing (76). These findings imply that while some IP subunits (PSMB9) are tumor suppressors, others (PSMB10) may have resistant, immune-evading stem-like reservoirs (**Figure 5**).

Other regulatory levels detail IP cross-interactions with immune checkpoints. In lung cancer, IL-6/STAT3-inducible USP24 stabilizes PD-1 protein by deubiquitinating K48-linked ubiquitin chains; USP24 inhibition suppresses T-cell exhaustion and restores immunotherapy responsiveness, implicating IP-regulated deubiquitination in immune escape (83). Furthermore, in CRC, overexpression of MCL-1, though traditionally anti-apoptotic, was paradoxically associated with favorable immune characteristics (PD-L1, MSI-H, TMB-high, inflamed TME). In contrast, inhibition of MCL-1 re-sensitized resistant TNBC/IBC models to mitogen-activated protein kinase kinase (MEK) inhibitors, pinpointing IP-related survival mechanisms as drug vulnerabilities (77, 78). A final complexity arises in vaccine contexts: leukemia-infected cell vaccines (ICVs) have shown that IP induction by oncolytic viruses is not essential for therapeutic efficacy (79). This highlights that in certain immunotherapy modalities, IP presence may be redundant, while in others it remains indispensable for epitope diversification and T-cell priming.

**Figure 6:**
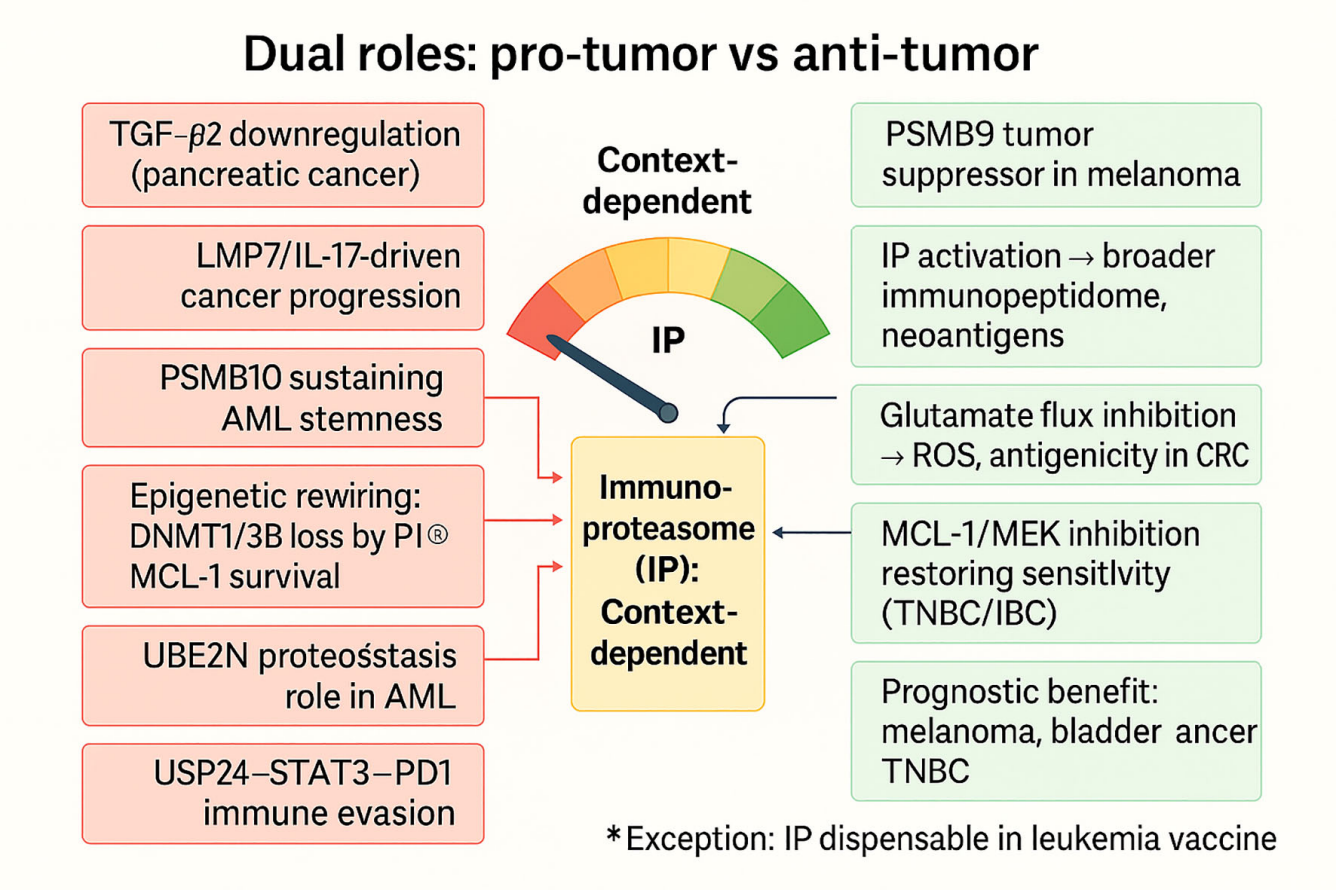
Context-dependent dual roles of the immunoproteasome in cancer. The immunoproteasome (IP) exerts divergent functions depending on tumor type and microenvironmental context. On the pro-tumor side (red), IP activity sustains chronic inflammation, promotes IL-17/LMP7-driven prostate cancer progression, maintains AML stemness (PSMB10, UBE2N), rewires epigenetic and metabolic programs (DNMT1/3B loss, MCL-1 survival), and supports immune evasion via the USP24–STAT3–PD1 axis. Conversely, in immunogenic contexts (green), IP enhances tumor suppression by broadening the immunopeptidome and unmasking neoantigens, promoting CD8^+^ T-cell responses, with PSMB9 acting as a tumor suppressor in melanoma, glutamate flux inhibition boosting antigenicity in CRC, and dual MCL-1/MEK inhibition restoring therapy sensitivity in TNBC/IBC. High IP expression aligns with favorable prognosis in melanoma, bladder cancer, and TNBC. Notably, IP is dispensable for leukemia infected-cell vaccine efficacy (exception). Collectively, these findings position the IP as a context-dependent regulator, balancing between tumor-promoting inflammation and antigen-driven immune surveillance.

Collectively, the dual roles of the immunoproteasome reflect a context-dependent balance between inflammatory protumor support and antigenic antitumor immunity (**Figure 6**). These contrasting functions are schematically summarized in **Figure 5**, which illustrates context-dependent pro-tumor mechanisms (e.g., AML stemness, IL-17-driven progression, proteostasis support) versus anti-tumor effects (e.g., PSMB9 tumor suppression, neoantigen diversification, enhanced immunogenicity), highlighting the immunoproteasome’s dualistic role in cancer biology. In inflammatory tumors (CAC, CRPC, pancreatic cancer, AML), IP sustains cytokine-driven progression and proteostasis, while in immunogenic contexts (melanoma, MSI-high CRC), it empowers immune recognition and therapeutic response. Subunit-specific functions (PSMB9 as a suppressor *vs*. PSMB10 as a stemness-maintainer) and regulatory axes (UBE2N, USP24, TGF-β2) add further granularity. For clinical translation, this dichotomy demands a precision strategy. In tumors where IP amplifies harmful inflammation or checkpoint stabilization, inhibition (e.g., ONX-0914, USP24 blockade, TGF-β2 targeting) may be therapeutic, whereas in tumors where IP augments antigenicity, its induction or preservation could be leveraged as a biomarker or treatment adjuvant. Thus, the immunoproteasome emerges not as a uniformly beneficial or detrimental factor, but as a dynamic regulator whose manipulation must be tuned to tumor type, subunit profile, and immune–metabolic context.

## 4 Cancer-type–specific immunopeptidomes

The following sections focus on a subset of cancer types, colorectal, breast, hepatocellular, lung, glioblastoma, and prostate, that collectively represent the most mechanistically characterized and immunopeptidomically mapped entities to date. These were selected because they exemplify distinct principles of antigen visibility, including genomic sparseness (CRC), heterogeneity (BC), viral integration (HCC), translational control (lung), immune privilege (GBM), and hormonal regulation (PCa). Together, they capture the conceptual diversity of how the tumor immunopeptidome is shaped across oncologic contexts, rather than aiming for exhaustive disease coverage.

The landscape of tumor immunopeptidomes is highly context-dependent, with each cancer type exhibiting distinct mechanisms that govern antigen visibility and therapeutic exploitability. Though colorectal cancers describe antigen paucity, breast cancer represents heterogeneity between antigen-plentiful TNBC and presentation-limited HR^+^ disease. Hepatocellular carcinoma integrates viral- and tumor-derived epitopes, whereas lung cancer highlights the dominance of noncanonical antigens shaped by translational control. Glioblastoma, despite its immune-cold reputation, reveals microbial mimicry and therapy-induced remodeling, and prostate cancer illustrates androgen-driven repression of antigen presentation. Combined, these paragraphs demonstrate the range in which immunopeptidomes are lean, suppressed, diversified, or reprogrammed. An overview of outstanding studies, techniques, and therapeutic significance is presented in **Table 2**, providing an introduction to the exploration of cancer-specific subsections.

**Table 2:** Summary of cancer-type–specific immunopeptidomes across colorectal, breast, liver, lung, brain, and prostate tumors, highlighting cohorts, technologies, and therapeutic implications.

Cancer type	Cancer subtype/cohort	Technology/approach	Antigen source category	Key findings (quantitative)	Therapeutic implication	Ref
Colorectal Cancer	CRC organoids	LC-MS/MS immunopeptidomics	Neoantigen landscape	Sparse HLA-I neoantigen landscape; no increase with IFNγ or MEKi	Limited utility of canonical neoantigen targeting	(124)
Colorectal Cancer	Colon cancer cell lines	Proteasome inhibition, signaling assays	STAT1/MHC-I modulation	Proteasome inhibition restored STAT1 and MHC-I expression	Combination with ICB	(125)
Colorectal Cancer	CRC patient samples	FAIMS mass spectrometry	Driver mutation neoantigens	Identified KRAS-G12V and other driver ligands	Neoantigen vaccine targets	(126)
Colorectal Cancer	Colon cancer cell lines	Drug perturbation + immunopeptidomics	Topoisomerase inhibition	Altered PD-L1 and MHC-I via DDR/NF-κB	Synergy with ICB	(127)
Colorectal Cancer	Colon cancer cells	Drug-induced immunopeptidomics	Topo-inhibitor induced peptides	83% expansion of ligands; unique/recurrent clusters	Drug priming for immunotherapy	(128)
Colorectal Cancer	CRC MSI-H vs MSS	Comparative immunopeptidomics	MSI-status neoantigens	MSI-H enriched in frameshift peptides	Prioritize MSI-H vaccines	(129)
Colorectal Cancer	Colon cancer cells	IFNγ stimulation	Cytokine-driven repertoire	Depletion of proline-rich epitopes under IFNγ	Context-aware antigen design	(42)
Colorectal Cancer	Metastatic CRC	Oncofetal peptide profiling	Oncofetal antigens	Fetal program peptides identified	Oncofetal vaccine/TCR targets	(130)
Colorectal Cancer	CRC tumors	Proteogenomics + microbiome	Tumor + microbial peptides	Neoantigens + bacterial ligands identified	Microbiome-integrated immunotherapy	(131)
Colorectal Cancer	CRC tumors	Immunopeptidomics	Microbial-derived peptides	Fusobacterium peptides activated CD8+ T cells	Microbiome-based immunotherapy	(132)
Colorectal Cancer	Multi-cancer incl. CRC	ESCAPE-seq DNA sequencing	Shared epitopes	>75,000 peptide-HLA interactions; recurrent epitopes	Shared epitope prioritization	(133)
Breast Cancer	Primary BC (HR+ & TNBC)	LC-MS/MS immunopeptidomics; proteogenomics; T-cell assays	Shared TSAs/TAAs	57,094 ligands; 25 TSAs; TSA burden ↑ with leukocytes & OS	Population-level TSA targeting	(134)
Breast Cancer	Pan-cancer incl. BC	Re-analysis; peptide validation	PTSPs	<3.1% HLA-I; <0.5% HLA-II; recurrent	Curated PTSPs as shared targets	(47)
Breast Cancer	BC TMAs (HR+/HER2+/TNBC)	Allele-resolved IHC	Allele-specific HLA loss	Subtype-biased loss (HR+, HER2+, TNBC)	Allele-aware vaccine stratification	(135)
Breast Cancer	TNBC models	ATM inhibition; DDR→APM signalling	APM boosting	MHC-I ↑; CD8 infiltration ↑; tumor ↓	Combine DDR modulators with immunotherapy	(136)
Breast Cancer	Murine BC (defined neoantigen)	MHC-II restricted immunity; serum transfer	CD4-B cell axis	Spontaneous rejection; humoral transfer	Include CD4-B axis in design	(137)
Breast Cancer	In silico PRAME+ BC	Immunoinformatics vaccine design	PRAME CT antigen	Predicted stable, antigenic vaccine construct	Candidate vaccine; validation needed	(138)
Breast Cancer	MCF7 & xenografts	Birinapant + immunopeptidomics	CTA & indel-derived peptides	↑ ligands; validated indel neoantigen; ↑ immunogenicity	Drug-induced peptidome reshaping	(43)
Lung Cancer	LUAD mouse model	*In vivo* MHC-I immunopeptidomics	Tumor peptides	Novel LUAD peptides; CD8 T responses	Supports antigen discovery	(139)
Lung Cancer	Lung cancer lines	Ribosome profiling + MS	nuORF peptides	Cap-independent translation induced peptides	Translational stress as immunomodulator	(140)
Lung Cancer	Population cohorts	Genetics + scRNA	HLA-II heterozygosity	HLA-II heterozygosity protective; LOH in tumors	Biomarker for risk stratification	(141)
Lung Cancer	Lung tumors	Immunopeptidomics + spatial transcriptomics	Neoantigens inflamed vs cold	Neoantigen hotspots enriched in immune-excluded tumors	Therapy tailoring to immune context	(142)
Lung Cancer	Pan-cancer incl. lung	Large-scale immunopeptidomics + ML	Canonical + noncanonical peptides	389k canonical; 70k noncanonical; validated shared neoantigens	Atlas + ML pipeline for prioritization	(143)
Lung Cancer	Lung cancer vaccine mice	Bioinformatics + immunization	Mutant Ddx21 peptide	Vaccine induced memory T cells, antitumor immunity	Prophylactic vaccine candidate	(144)
Lung Cancer	SCLC, HLA-A*0201+	Immunopeptidomics + IHC	ATAD2 peptide	Epitope identified; correlated with ASCL1	TCR/ACT target for SCLC	(145)
Lung Cancer	NSCLC + melanoma	Proteogenomics	Unmutated aeTSAs	589 TAs; 99% from unmutated sequences	aeTSA as immunotherapy targets	(146)
Lung Cancer	Lung (LLC mice)	Translatome + MS	Translatome-derived antigens	10 neoantigens identified; 8 immunogenic	Peptide vaccine candidates	(147)
Lung Cancer	NSCLC tumors	IHC + imaging	NK/CD8 infiltration	High NK/CD8 improved survival despite MHC-I loss	Synergistic NK+CD8 therapy	(148)
Lung Cancer	NSCLC patients	Proteogenomics + MS	Mutant neoantigens	1 identified; validated responses in 5/6 pts	Neoantigen vaccine development	(149)
Prostate Cancer	PCa mouse model	MHC-I PSA tetramer	PSA peptides	Validated PSA tetramer detects CD8 T cells	Tool for PSA vaccine evaluation	(150)
Prostate Cancer	PCa cell lines + DCs	DC vaccine (MAGE-A2 peptide)	MAGE-A2 antigens	Induced CTLs with strong cytotoxicity	Basis for PCa DC vaccine	(151)
Prostate Cancer	CD4 T cell platform	EliteMHCII profiling	MHC-II epitopes	High affinity epitopes correlate with CD4 T responses	Epitope discovery tool	(152)
Prostate Cancer	Prostate cancer models	AR inhibition + CRISPRi	MHCI regulation	AR represses MHCI; blockade restores expression	Target AR to improve antigen presentation	(153)
Prostate Cancer	Humans + HLA-DR4 mice	Epitope screening + T cell assays	PSMA459 epitope	PSMA459 induced CD4 responses	Candidate Th epitope for vaccines	(154)
Prostate Cancer	PCa cell lines + GILT	GILT insertion to enhance HLA-II	PSMA459 presentation	GILT increased CD4 activation; cysteinylation inhibited	GILT-based vaccine enhancement	(155)
Glioblastoma	GBM tissues + cell lines	HLA immunopeptidomics	Microbial + tumor peptides	Bacterial peptides presented; recognized by TILs	Microbial peptides as vaccine targets	(156)
Glioblastoma	In silico GBM vaccine	Epitope prediction + docking	CD204 TAM epitopes	Multi-epitope vaccine design validated computationally	Candidate peptide-based GBM vaccine	(157)
Glioblastoma	GBM proteins (EGFR, IDH1, PTEN, TP53)	IEDB + modeling + dynamics	Mutant vs WT epitopes	Mutations did not disrupt epitope presentation	Supports vaccines targeting GBM mutations	(158)
Glioblastoma	Mouse GBM (GL261-CIITA)	CIITA-driven MHC-II expression	CIITA-induced tumor antigens	GL261-CIITA vaccination rejected tumors; immune memory	Proof-of-concept GBM vaccine	(159)
Glioblastoma	GBM patients treated with CAN-3110	Serial immunopeptidomics	Dynamic TAA presentation	CAN-3110 increased HLA-I/II; altered repertoire	Oncolytic virus remodels GBM immunopeptidome	(160)

### 4.1 Colorectal cancer immunopeptidome

The immunopeptidome of CRC presents a paradox, characterized by a high mutational load in some subtypes, yet consistently sparse antigenic visibility at the HLA surface. Early work using patient-derived organoids (PDOs) demonstrated that, across 612 non-silent mutations, only three HLA-I ligands were directly detected, representing 0.5% of the mutational burden, and no additional epitopes were uncovered even after IFNγ or MEK inhibitor treatment (124). This establishes CRC, particularly microsatellite-stable (MSS) tumors, as profoundly antigen-poor despite genomic diversity. Mechanistic research has implicated defective STAT1 signaling as a mechanism of this invisibility. MSS CRC cells and tumor tissues exhibited defective STAT1 activation in response to IFN-γ stimulation, characterized by extremely low induction of MHC-I and PD-L1 (125). Pharmacological inhibition of the proteasome with bortezomib restored STAT1 phosphorylation, rescued MHC-I expression, and enhanced T-cell visibility (125). This implies that defects in cytokine signal transduction pathways upstream of antigen presentation are targetable therapeutic targets.

More refined approaches have enabled the direct detection of driver-derived epitopes in CRC. Using differential ion mobility mass spectrometry, investigators detected KRAS-G12V and CPPED1-R228Q neoantigens bound to HLA-I in tumor samples, validating their immunogenicity experimentally (126). This proof-of-concept demonstrates how cutting-edge technologies can overcome the limitations of prediction pipelines, which often overestimate the immunogenic repertoire. Cytotoxic drug exposure remodels the CRC immunopeptidome. Topoisomerase inhibition altered the surface landscape of PD-L1 and MHC-I through DNA Damage Repair (DDR) and NF-κB signaling (127). Global immunopeptidomic profiling revealed an 83% expansion of MHC-I ligands under drug treatment, including both unique and recurrent clusters, compared to untreated cells (128). Importantly, whether these drug-induced peptides contribute to T-cell immunogenicity remains to be clarified.

The microsatellite instability (MSI) function has also been further studied through comparative peptidomics. Frame-shift derived ligand-enriched MSI-high cancers, but MSS tumors remained free of new epitopes, highlighting the immunological gap between subtypes (129). Simultaneously, remodeling induced by IFNγ was observed to globally remove proline-containing epitopes, independently of protein abundance, highlighting that peptide chemistry serves as the sole selective filter (42). Aside from coding regions, proteogenomics identified tumor antigens of noncoding origin, like cryptic ORFs and translation of noncoding RNA, increasing the universe of epitopes for MSI and MSS tumors (129). A metastatic CRC fetal-like transcription program also yields oncofetal peptides that are not expressed in normal adult tissue but are immunoselectively effective (130).

Microbial contributions constitute yet another novel axis. Fusobacterium nucleatum-positive CRC tumors contained bacterial-derived ligands in the process of engaging autologous CD8+ T cells (131). Additional analysis verified the presence of numerous microbial peptides in the tumor immunopeptidome, which may be potential targets for immunotherapeutic manipulation (132). These findings indicate the co-ordination of tumor genetics, developmentally regulated programs, and microbial ecology in defining the antigenic landscape of CRC. Population-scale discovery studies have advanced the scale of CRC immunopeptidomics beyond that of individual case studies. ESCAPE-seq enabled the screening of more than 75,000 peptide–HLA pairs and revealed that common epitopes of CRC driver mutations are present on numerous alleles, with an approximate global coverage of 90% (133). These findings highlight the fact that the CRC immunopeptidome is not fixed but a pharmacologically manipulable repertoire.

**Figure 7:**
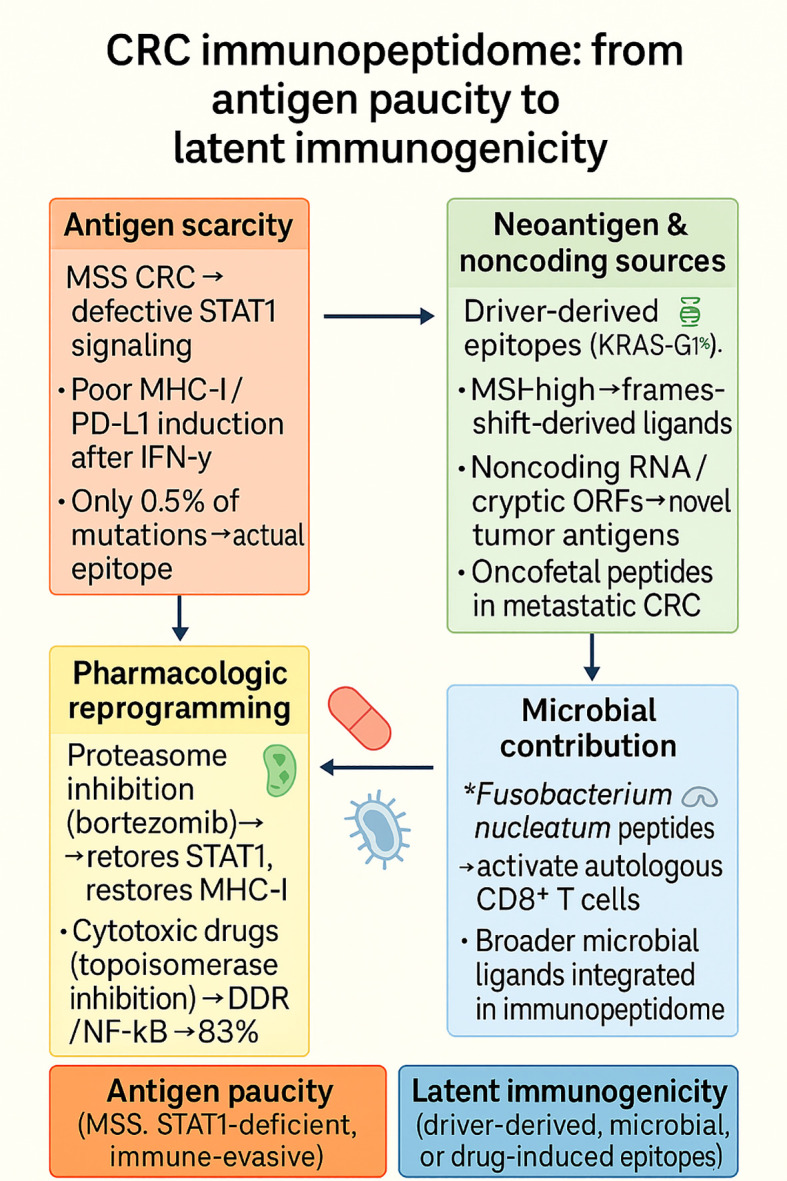
The CRC immunopeptidome is characterized by profound antigen paucity in MSS tumors due to defective STAT1 signaling and poor IFN-γ–induced MHC-I expression, but can be pharmacologically reprogrammed (e.g., by proteasome or cytotoxic drugs) to restore antigen visibility. Beyond this scarcity, latent immunogenicity emerges from driver-derived epitopes (e.g., KRAS-G12V), noncoding ORFs, oncofetal peptides, and microbial ligands such as Fusobacterium nucleatum, highlighting both constraints and opportunities for therapeutic exploitation.

In summary, these studies establish a consensus position: the CRC immunopeptidome is inherently parsimonious for normal neoantigens, particularly in MSS cancers, but can be rewritable through cytokine signaling manipulation, pharmacological interference, proteogenomic discovery, and microbial hijacking. These multilayered paucity and rediscovery cycles of antigen are represented schematically in **Figure 7**, which illustrates how MSS CRC is inherently antigen-depleted but susceptible to reprogramming by pharmacologic, proteogenomic, developmental, and microbial stimuli. The story is shifting away from antigen poverty and toward a theme of latent immunogenicity, in which cryptic, driver-derived, noncoding, developmental, or microbial epitopes are poised to be unveiled in a coordinated fashion. These results redefine CRC as a tumor entity where the secret is not that it is target-deficient but lies in the discovery and utilization of as yet unexplored reserves of its immunopeptidome.

### 4.2 Breast cancer immunopeptidome

BC displays remarkable immunologic heterogeneity, TNBC usually has an inflamed microenvironment, while HR^+^ tumors are generally “cold” (161). Direct immunopeptidomic mapping now reveals that both subtypes share numerous tumor antigens (TSAs) that largely do not originate from classical coding mutations. In 26 primary BCs, mass spectrometry identified 57,094 unique MHC-I–associated peptides (MAPs) and 25 TSAs, most originating from aberrantly expressed regions rather than somatic mutations. TSAs were more frequently observed in TNBC than HR^+^ disease, and the predicted TSA burden positively correlated with leukocyte infiltration and overall survival in TNBC, consistent with *in vivo* immunogenicity. Notably, 49 TAAs—including peptides arising from cancer-associated fibroblasts were also detected, and several TSAs/TAAs elicited antigen-specific T-cell responses *in vitro*, indicating a rich, shared antigenic landscape suitable for population-level targeting (134).

A second, qualitatively distinct antigen source is post-translationally spliced peptides (PTSPs). Re-analysis of a significant cancer peptidome resource with a stringent pipeline found PTSPs constitute <3.1% of HLA-I ligands and <0.5% of HLA-II ligands overall. Although numerically modest, PTSPs were recurrent across samples and included products from cancer/immune genes (e.g., MITF, DAPK1, HLA-E), with synthetic-peptide validation and evidence of immunogenicity. The inference for BC is that PTSPs add diversity and recurrency on top of the dominant non-mutant TSA space—an attractive niche for shared-antigen vaccines, provided that analytical false positives are carefully controlled (47). Antigen visibility ultimately depends on HLA expression and allelic integrity. An allele-resolved IHC framework was validated using pan- and allele-specific antibodies to quantify HLA-A/B/C loss *in situ*, and then applied to breast tumors. The study uncovered subtype-specific patterns: higher HLA-A/B loss in hormone-driven cancers, preferential HLA-B loss in HER2^+^ tumors, and balanced loss of A/B/C in TNBC. Notably, HLA-A/B loss appeared as an early event in premalignant lesions, whereas HLA-C loss was less frequent throughout evolution. These data suggest that trial designs predicated on antigen-specific T cells (checkpoint inhibitors, vaccines, TCR therapies) should pre-screen for allele expression, as allele-specific loss could silently limit efficacy in otherwise antigen-rich tumors (135).

Mechanistically, DDR signaling can be leveraged to restore HLA visibility. In TNBC, ATM inhibition increased MHC-I expression through a c-Jun/TNF-α/p-STAT1 axis, augmented CD8^+^ T-cell infiltration and cytotoxicity, slowed tumor growth, and sensitized tumors to PD-1 blockade and radiotherapy *in vivo*. This places ATM as an immune-suppressive node whose inhibition converts TNBC toward a more “visible” phenotype and provides a rational combination partner for immunotherapies targeting the immunopeptidome (136). Complementing this, the IAP antagonist Birinapant (a SMAC mimetic) quantitatively and qualitatively reshaped the immunopeptidome *in vitro* (including MCF7 breast-cancer cells) and *in vivo*: it increased the number and abundance of class-I ligands and source proteins, enriched cancer-testis–antigen peptides and neoantigens, and provided functional evidence for a validated indel-derived neoantigen; in xenografts, Birinapant treatment similarly expanded HLA ligands and improved tumor immunogenicity, consistent with enhanced activity in combination with checkpoint blockade (43).

Beyond class I, class II immunity can independently control BC. In murine models bearing breast carcinomas engineered to express a defined neoantigen (rat-erbB2), spontaneous tumor rejection occurred in MHC-II–restricted settings and depended on CD4^+^ T cells, B cells, and antigen-specific antibodies. Passive transfer of immune serum conferred protection, and introducing the same neoantigen into other H-2^b^ tumor models preserved the rejection phenotype. While based on model antigens, these data underscore how MHC-II presentation and humoral immunity can drive tumor clearance—an axis worth integrating with class-I–centric strategies in BC (137). Finally, target nomination is beginning to translate into design heuristics. An immunoinformatics program focused on PRAME (a cancer-testis antigen expressed in subsets of BC) constructed a multi-epitope vaccine whose predicted properties included non-allergenicity, favorable hydrophilicity, and in-silico engagement of Toll-like receptor 4 (TLR4) and interleukin-1 receptor (IL-1R); codon optimization and cloning were shown computationally. While experimental validation remains essential, PRAME illustrates how shared, non-mutant antigens can be prioritized for vaccine constructs that complement (rather than replace) proteogenomically observed ligands (138).

Taken together, contemporary BC immunopeptidomics converges on four principles. First, the most actionable antigens are shared and non-mutant (aberrant-expression TSAs, TAAs), with PTSPs providing additional recurrent specificity. Second, allele-specific HLA loss is common and may be subtype-biased, arguing for allele-aware eligibility and endpoint analyses in trials. Third, DDR-APM cross-talk (e.g., ATM→c-Jun/TNF-α→p-STAT1) provides a drug-targetable lever to enhance MHC-I display and immunotherapy responsiveness. Fourth, the MHC-II/CD4-B-cell axis can mediate tumor rejection and should be considered in vaccine and cell-therapy designs, especially for HR^+^ tumors where class I visibility is limited. Current evidence reframes BC as antigen-rich but presentation-constrained. Actionable priorities are: (i) immunopeptidomics-first discovery emphasizing shared, non-mutant TSAs (with curated PTSPs); (ii) allele-aware patient selection and endpoints; (iii) APM boosting via DDR/IFN-pathway modulation; and (iv) purposeful inclusion of MHC-II/CD4–B-cell mechanisms. Together, these principles move BC immunotherapy beyond mutation load toward mechanistically grounded, antigen-precise interventions.

### 4.3 Hepatocellular carcinoma

HCC sits at the intersection of oncogenesis and chronic viral infection, and its antigenic landscape reflects both forces. Direct immunopeptidomic profiling of primary human hepatocytes isolated from hepatitis B virus (HBV)-infected non-tumor and HCC tissues revealed a vast HLA-I peptidome (~2×10^5 ligands) but only a scarce set of HBV-derived peptides (n=8) and tumor-associated antigen (TAA) ligands unique to tumors (14 peptides from 8 TAAs); targeted MS confirmed most candidates and functional assays validated immunogenicity for 5 HBV and 3 TAA peptides (162). The picture that emerges is one of abundant self-peptide display with punctate viral/tumor epitopes, implying that immune escape in HCC may derive less from a complete failure of antigen presentation and more from low-density, hard-to-detect targets —a constraint that rationalizes the modest performance of single-epitope vaccines and argues for multi-epitope designs.

A complementary line of evidence expands the target space beyond canonical coding regions. By integrating long-read/short-read RNA-seq with MS, recent work uncovered non-canonical antigens arising from cryptic translation of non-canonical open reading frames (ncORFs) in HCC: 12 peptides were identified, with four showing tumor-enriched expression and strong predicted MHC/T-cell receptor (TCR) binding; ribosome recruitment and m^6A-mediated initiation emerged as enabling mechanisms (163). These data suggest that HCC’s immunopeptidome encompasses previously uncharted ncORF epitopes, some of which are preferentially expressed in tumors, thereby expanding the repertoire for T-cell therapies and vaccines while highlighting a dependency on translation control and RNA modifications that could be co-targeted.

Translationally, antigen discovery must be paired with presentation-competent delivery. Immunopeptidomics of human dendritic cells pulsed with HBV-based synthetic long peptides (SLPs), a platform relevant to HBV-driven HCC, showed that TLR1/2 versus TLR3 adjuvants remodel the DC immunopeptidome without altering the qualitative ability to cross-present SLPs; 33 unique HLA-I peptides were directly detected, several missed by in-silico prediction, with a bias toward HLA-B presentation and donor-recurrent ligands (164). For HCC vaccine design, these findings underscore two imperatives: (i) empirical immunopeptidomics is needed to complement prediction, and (ii) adjuvant selection modulates the peptide landscape available to CD8^+^ T cells, a controllable variable in clinical formulations.

Finally, HCC, like other tumors, may harbor defects in the antigen-processing/presentation machinery (APPM) that selectively rewire what is displayed. A systematic CRISPR knockout survey across key APPM genes (e.g., B2M, TAP1/2, TAPBP, ERAP1, CALR, CANX) demonstrated allele-restricted and subset-specific losses in presented peptides, with marked changes when CALR/CANX or TAP1 were ablated (165). Although performed in an isogenic reference line, the implications for HCC are direct: epitope selection for vaccines or TCR therapies should favor ligands resilient to common APPM bottlenecks (e.g., TAP-light or chaperone-independent binders), and companion diagnostics should assess APPM integrity to stratify patients *a priori*.

The HCC immunopeptidome consists of (i) light immunogenic HBV and TAA ligands (162), (ii) a broadened ncORF-derived antigen layer with translation-biology dependencies (163), nd (iii) a peptide output that is tunable to vaccine adjuvants (164) but vulnerable to APPM lesions (165). A realist approach is a two-step, high-precision strategy: tumor-informed discovery (bulk and targeted immunopeptidomics with canonical and non-canonical sources, centered on the patient’s HLA type), followed by presentation-conscious development, utilizing dendritic cell (DC)-stimulating adjuvants that promote the selection of intended ligands and APPM-resistant epitopes. This paradigm redirects HCC immunotherapy from a search for “the” antigen to engineering a discernible, stable antigen set responsive to the molecular environment of every liver.

### 4.4 Lung cancer immunopeptidome

The lung cancers, especially NSCLC and the neuroendocrine small cell lung cancer (SCLC) variant, are extremely genomically heterogeneous, yet HLA-presented antigens are generated by processes well beyond simple mutation-to-epitope translation. *In vivo* trapping of HLA-I ligands in knock-in affinity tag (KbStrep) genetically engineered lung adenocarcinoma (LUAD) demonstrated that peptide presentation cannot be inferred from mRNA or translational levels; numerous immunogenic ligands from poorly expressed genes, and peptide hierarchies varied across the alveolar type-2 cell–to–late-stage continuum. Vaccination with *in-vivo*–eluted LUAD peptides elicited CD8^+^ responses in naïve and tumor-bearing mice, underscoring the physiological relevance of directly observed ligands (139). Spatially resolved multi-omic profiling of human lung tumors (61 regions; 8 patients) mapped the lung cancer immunopeptidome onto T-cell-inflamed *vs* T-cell–excluded niches. Predicted neoantigens were enriched within HLA-I “presentation hotspots” in T-cell–excluded regions; concordant evidence of immune recognition suggested ongoing immune editing and provided a rationale for microenvironment-tailored combinations (e.g., epitope-focused vaccines with checkpoint or myeloid-modulating agents) (142).

Antigen origin is frequently non-mutational. A pan-cancer atlas (531 samples) catalogued >459k peptides (≈389k canonical; 70,270 noncanonical), with noncanonical ligands presented at levels comparable to canonical peptides. A machine-learning pipeline (MaNeo) prioritized candidates and prospectively validated multiple neo-peptides, establishing an immunopeptidomics-guided route to clinical targets (143). In NSCLC and melanoma, proteogenomic dissection found that ≈99% of tumor antigens derived from unmutated sequences, including aberrantly expressed tumor-specific antigens (aeTSAs), overexpressed TAAs, and lineage-specific antigens, whereas only ~1% mapped to mutations; aeTSAs were often encoded by noncanonical sequences and were shared and immunogenic, reframing target selection beyond classic mutation-centric strategies (146). Translational control is a major determinant of antigen visibility. Inducing a shift from cap-dependent to cap-independent translation with the eukaryotic initiation factor-4A (eIF4A) inhibitor silvestrol selectively remodeled novel unannotated ORF (nuORF)-derived ligands, in sharp contrast to global translational blockade (homoharringtonine), which dampened presentation, implicating stress-responsive translation in shaping cryptic antigen display in lung cancer (140). Complementarily, translatome-first antigen discovery (ribosome-nascent-chain capture in Lewis lung carcinoma (LLC) cells) produced ten candidate neoantigens, eight of which proved strongly immunogenic *in vitro* and yielded *in vivo* vaccine efficacy, linking ongoing translation to actionable vaccine payloads (147).

On the population genetics axis, HLA-II heterozygosity is associated with a reduced lung-cancer risk in smokers, and tumor HLA-II loss of heterozygosity (LOH) favors the loss of alleles with larger neopeptide repertoires—implicating both inherited and somatic variation in antigen surveillance and selection (141). In the tissue ecosystem, despite frequent MHC-I loss in NSCLC, spatial multiplex imaging revealed that NK–CD8^+^ codensities and IFNγ^+^ lymphocyte neighborhoods co-localized with MHC-I^+^ tumor islands and were associated with improved survival, suggesting that NK–T-cell coordination serves as a compensatory axis when antigen presentation is regionally impaired (148). At the single-target level, SCLC immunopeptidomics identified an HLA-A*02:01–restricted epitope from ATAD2 (YSDDDVPSV) with robust T-cell recognition, offering a subtype-specific, shared target for adoptive and vaccine strategies (145). In prophylactic settings, a Ddx21 mutant peptide generated durable anti-tumor immunity and central-memory T-cell expansion in murine lung-cancer vaccination, suggesting preventive or adjuvant opportunities when high-risk lesions are identifiable (144). Finally, patient-level proteogenomics in NSCLC demonstrated that filtering predictions through the observed immunopeptidome increased functional hit rates (13% overall; clonal TCR expansion tied to a class-I ligand in one case), supporting immunopeptidomics-gated pipelines for individualized vaccines (149).

**Figure 8:**
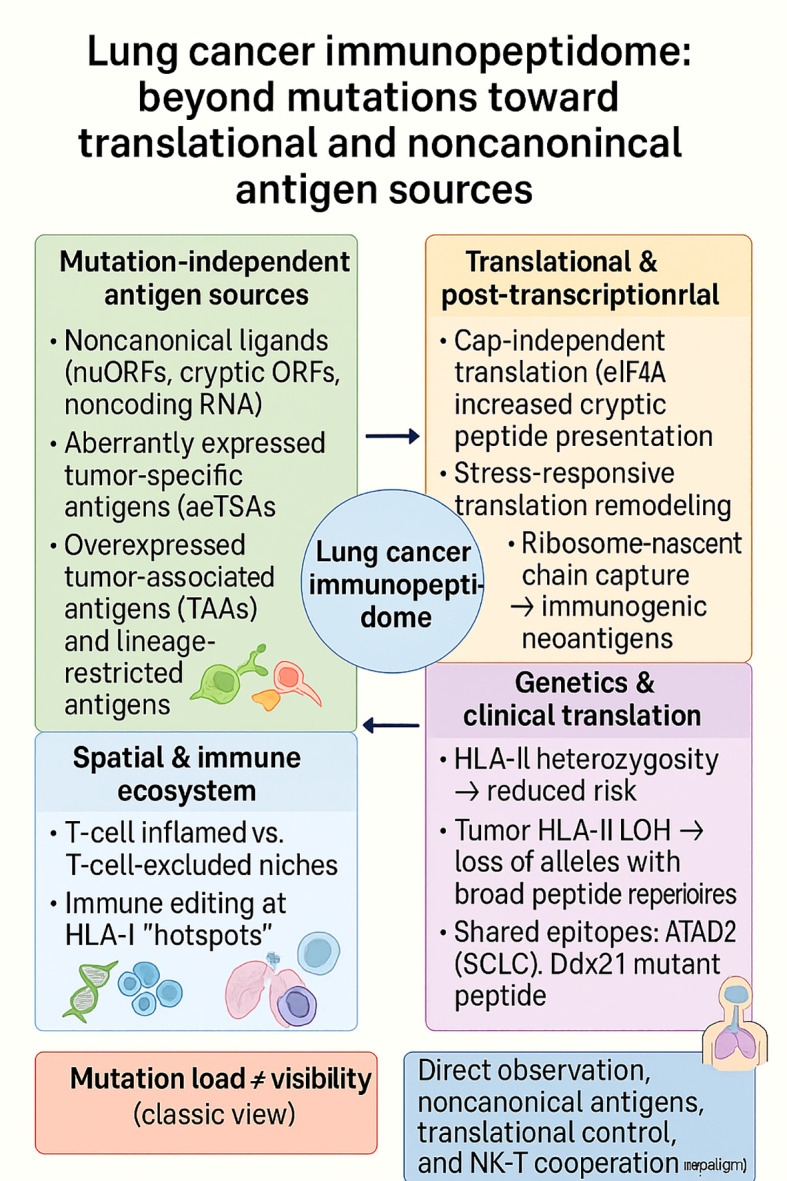
The lung cancer immunopeptidome extends beyond mutational burden, integrating noncanonical and lineage-restricted antigens, translational remodeling, spatial immune niches, and HLA variation. These context-dependent layers explain why mutation load does not equate to antigen visibility and highlight new therapeutic paradigms centered on direct ligand observation, noncanonical antigen discovery, translation-aware modulation, and NK–T cell cooperation.

In LUAD, squamous NSCLC, and SCLC, presentation rather than mutation controls immunogenic visibility. Lung cancers expose antigens through post-transcriptional and translational control (nuORFs, noncanonical/lineage programs), are spatially confined to alter immunity, and are also conditioned by germline/LOH variation at HLA. These various determinants are diagrammatically illustrated in **Figure 8** and indicate how mutation-independent sources of antigens, translational remodeling, spatial–immune niches, and HLA variation collectively determine the lung cancer immunopeptidome, in addition to mutation load. The ensuing therapy is immunopeptidomics-first: it monitors ligands *in situ in vivo*, targets noncanonical and shared aeTSAs, leverages translation-aware modulation, and benefits from NK–T-cell collaboration in areas of MHC-I loss. This convergence advances lung-cancer immunotherapy from mutation burden to mechanistically directed, antigen-specific therapies.

### 4.5 Glioblastoma immunopeptidome

GBM has long been regarded as an “immune-cold” tumor, partly due to its highly suppressive tumor microenvironment and its sanctuary within the central nervous system (166, 167). However, recent immunopeptidomic analyses have banished such a notion by showing that GBM is not only antigenically complex but also that its peptide landscape is restricted by low density and context-dependent presentation. In a surprising finding, the GBM immunopeptidome also contained bacterial peptides, which were isolated from tumor samples and cell lines. Tumor-infiltrating T cells had seen such HLA-II–restricted microbial epitopes, implicating a molecular mimicry process whereby T cells activated against commensals or pathogens cross-react with glioblastoma ligands. While such mimicry heightens immunogenicity, it does so at the expense of potential off-target autoimmunity, which shows the double-edged nature of cross-priming in GBM (156).

Aside from exogenous peptides, analysis of repeat GBM mutations showed that persistence of neoantigens is stronger than expected. Structural modeling of tumor protein p53 (TP53), isocitrate dehydrogenase 1 (IDH1), phosphatase and tensin homolog deleted on chromosome ten (PTEN), and epidermal growth factor receptor (EGFR) epitopes showed that most mutations did not eliminate MHC-I binding or CD8^+^ entry, maintaining recognition under the tolerance of sequence difference (158). These data indicate that despite the “neoantigen poor” status of GBM, certain epitope subsets are structurally intact and available for therapeutic targeting.

A second strategy to enhance GBM antigenicity has involved targeting class II expression by tumors. By using genetic modification to induce the expression of MHC Class II transactivator (CIITA), the MHC-II regulatory factor, researchers created highly immunogenic GL261-CIITA vaccines. They induced strong CD4^+^ and CD8^+^ infiltration and even provided protection against contralateral tumors across the blood–brain barrier, providing proof-of-principle that enhancement of class II presentation would induce long-lasting immunity in GBM (159). Parallel bioinformatic strategies have developed multi-epitope vaccine candidates against TAM-associated epitopes, such as CD204, to induce CTL, helper, and B-cell activity on various HLA alleles. In silico, the polyepitope vaccines tightly interacted with class I and II molecules and TLRs, predicting multi-axis immune activation (157).

Most importantly, the GBM immunopeptidome is dynamic and responsive to treatment. In tumor serial sampling following oncolytic virus CAN-3110 treatment, there was a striking upregulation of HLA-I and HLA-II ligands weeks after treatment, in addition to interferon signatures and new TAAs and cancer testis antigens (CTAs) emerging. These findings suggest that GBM’s antigenic profile is dynamic rather than static and can be reprogrammed pharmacologically, establishing temporal windows of augmented visibility that can be correlated with immunotherapeutic treatment (160).

Taken together, the GBM immunopeptidome is not silent but densely layered, incorporating tumor-restricted CTAs, microbial mimicry, resilient neoantigens, and inducible class II ligands. Its therapeutic exploitation requires moving beyond static antigen discovery toward context-aware and dynamic strategies: combining liquid-biopsy immunopeptidomics for biomarker tracking, leveraging microbial cross-reactivity with caution, enforcing class II presentation to sustain memory, and synchronizing vaccination or checkpoint blockade with therapy-induced bursts of antigen display. Such a framework redefines GBM immunotherapy from the pursuit of a single “privileged” epitope to the engineering of a durable, multi-epitope repertoire matched to temporal and microenvironmental context.

### 4.6 Prostate cancer immunopeptidome

Prostate cancer remains a paradigmatic “immune-cold” tumor in part because androgen signaling intersects the antigen-presentation machinery at multiple nodes. A genome-scale CRISPRi screen and orthogonal *in vivo* models identified the androgen receptor (AR) as a direct repressor of the MHC-I pathway; AR blockade transiently increased MHC-I expression, augmented T-cell–mediated tumor control, and suggested a therapeutic window in which AR inhibition could be paired with checkpoint blockade to convert low-visibility lesions into immunologically legible targets (153). Within this framework, the prostate immunopeptidome should be considered plastic and druggable, with AR activity acting as a tunable dial on epitope density.

On the CD8^+^ axis, the earliest translational efforts centered on prostate-specific antigen (PSA) as a canonical TAA. A murine H-2L^d^-restricted PSA peptide (HPQKVTKFML^188-197^) enabled the first PSA tetramer capable of directly enumerating PSA-specific CD8^+^ T cells elicited by a PSA-encoding adenoviral vaccine, moving beyond functional assays to quantitative tracking of vaccine-induced clones (150). This tool bridged antigen processing to measurable immune readouts and remains conceptually important: immunopeptidome-aware vaccine trials require direct pharmacodynamic biomarkers of epitope-specific CD8^+^ cells, not just tumor shrinkage.

Durable antitumor immunity, however, typically depends on CD4^+^ T-cell help and class II presentation. Work on prostate-specific membrane antigen (PSMA) mapped naturally processed, MHC-II–restricted helper epitopes, notably PSMA459 (NYTLRVDCTPLMYSL), which activated human CD4^+^ T cells across multiple HLA-DR alleles and primed responses in HLA-DR4 transgenic mice, evidence that class II ligands can be leveraged to sustain CTL responses and memory in PCa (154). Yet biochemical reality complicates epitope utility: cysteinylation of PSMA459 under physiologic cystine blunted CD4^+^ recognition, whereas engineering tumor cells to express GILT (a lysosomal thiol reductase) enhanced class II processing and restored helper responses, pinpointing redox-dependent peptide chemistry as a gatekeeper of the class II immunopeptidome in PCa (155). These findings suggest that helper-epitope selection and design should consider PTM liabilities (e.g., cysteinylation) and, where possible, stabilize intracellular processing barriers (e.g., through GILT).

Broadening beyond PSMA/PSA, DC vaccines pulsed with long peptides from MAGE-A2 drove robust T-cell expansion, IFN-γ production, and cytotoxicity against PCa lines (PC3, LNCaP), supporting the premise that long-peptide vaccines exploit endogenous processing to seed both class I and class II pathways and diversify the presented repertoire (151). Methodologically, CD4 epitope discovery has been limited by MS sensitivity and variable prediction accuracy; a high-throughput class II profiling platform (EliteMHCII) spanning 24 common alleles increases the throughput and allele coverage of helper-epitope mapping and provides a scalable route to chart the PCa class II immunopeptidome across global HLA diversity (152). Together, these studies converge on a practical theme: next-generation PCa vaccines should be multi-epitope, helper-competent, and immunopeptidome-verified.

Synthesis across these lines of evidence suggests a presentation-aware treatment logic for PCa. Pharmacologically, AR inhibition can transiently increase MHC-I levels and sensitize tumors to CTL-mediated control; however, this effect is time-limited and should be synchronized with checkpoint blockade or vaccine-driven T-cell expansion (153). Antigenically, helper-epitope inclusion (e.g., PSMA459-like sequences) improves CD8^+^ durability, but peptide chemistry and intracellular redox must be engineered to avoid PTM-mediated immune silencing (e.g., cysteinylation mitigated by GILT or by peptide design) (154, 155). From a measurement standpoint, tetramers (PSA and beyond) and class II discovery platforms should be built into trials as pharmacodynamic anchors to verify that the intended immunopeptidome is actually displayed in patients (150, 152). Conceptually, PCa’s “coldness” is not immutable; the tumor’s antigenic visibility can be dialed up by aligning androgen signaling control, helper-competent epitope design, and empirical immunopeptidomics.

## 5 Therapeutic implications: turning maps into medicines

### 5.1 Vaccines

Immunopeptidome maps are beginning to specify what to vaccinate against, how to formulate vaccines, and where to deploy them across the disease continuum. Three complementary lines of evidence —patient-derived target nomination, prophylactic efficacy with a single neoantigen, and platform-level optimization of DC cross-presentation—outline a practical roadmap from ligandomic discovery to clinical vaccines. A proteogenomic study in CRC integrated immunopeptidomics, whole-exome sequencing (WES), and 16S ribosomal DNA (rDNA) profiling across eight patients, directly identifying both tumor neoantigens and bacterial immunopeptides as vaccine-ready candidates (131). Autologous T-cell assays confirmed recognition of all neoantigens and 5/8 bacterial peptides, while TCR-αβ sequencing traced the repertoire of epitope-reactive CD8^+^ T cells; engineered TCR-T cells were activated by peptide-pulsed lymphoblastoid cells. Together, these data argue that (i) observed ligands, not only predictions, should seed payloads; (ii) microbial peptides represent an orthogonal, immunogenic class that may bypass central tolerance in CRC; and (iii) early-phase trials can prespecify TCR tracking and functional reactivity as on-target pharmacodynamic endpoints (131).

In lung cancer, immunization with a Ddx21 mutant peptide (Ddx21^MT) generated durable anti-tumor immunity and increased central memory (T_CM) CD8^+^ T cells in mice, explaining the superior protection previously seen with an early-lesion–derived vaccine (144). The lesson for translation is twofold: first, lesion chronology matters; neoantigens unique to early trajectories may be especially prophylactic; second, quality of the epitope (memory imprinting, breadth of presentation) can trump quantity. A rational trial niche is high-risk cohorts (e.g., heavy smokers with indeterminate pulmonary nodules) where a short epitope set derived from observed or shared early drivers could be evaluated for prevention or post-resection relapse control (144).

For therapeutic DC vaccines, immunopeptidomics of human DCs pulsed with 12 synthetic-long peptides (SLPs) was compared between TLR1/2 (Amplivant) and TLR3 (poly I:C) adjuvants, and the HLA-I ligandomes actually displayed to T cells were mapped (164). Thirty-three SLP-derived class-I peptides were identified, including ligands not predicted in silico—with a striking HLA-B bias in (cross-)presentation; adjuvants remodeled the DC immunopeptidome but did not materially change SLP cross-presentation efficiency. These results provide actionable heuristics: prioritize SLPs empirically observed on DCs, weight allele coverage toward HLA-B, and select adjuvants for immune-context tuning rather than for cross-presentation per se (164).

In practice, a Phase I/II workflow could: (i) run MS-first discovery with proteogenomics to nominate tumor and microbial ligands (CRC paradigm) (131); (ii) assemble a hybrid payload (patient-specific ligands + a small panel of shared/early neoantigens) with DC-validated SLPs emphasizing HLA-B coverage (164); (iii) vaccinate in the neoadjuvant/MRD window, with TCR sequencing, ex vivo cytotoxicity, and on-treatment immunopeptidomics as readouts; and (iv) consider prophylactic arms in high-risk cohorts using early-trajectory neoantigens (Ddx21^MT-like) (144). Combination with checkpoint blockade and with antigen-visibility modulators (e.g., IFNγ, DDR-pathway drugs) can be pre-specified where HLA display is limiting. Immunopeptidome maps are no longer descriptive; they determine vaccine content, platform, and timing. By coupling observed ligands (including microbial epitopes) (131), window-specific neoantigens that imprint memory (144), and empirically validated SLP/DC presentation rules (164), vaccine programs can move from “best-guess” to antigen-precise interventions with measurable on-target biology.

### 5.2 Checkpoint synergy & remodeling

Immune checkpoint blockade can only work if tumors are visible to T cells. The most consistent route to visibility is to remodel the antigen-presentation axis so that more and more “foreign”—HLA–peptide complexes are displayed precisely when PD-1/PD-L1 or cytotoxic T-lymphocyte–associated antigen 4 (CTLA-4) are inhibited. Across recent studies, a coherent picture emerges: re-establishing IFNγ–STAT1–APM signaling to raise HLA-I, anticipating IFNγ-driven repertoire shifts when selecting epitopes, scheduling DNA-damage–related drugs to increase rather than suppress display, and using IAP antagonism to expand the ligandome, including CTAs and bona fide neoantigens.

In colorectal cancer models with low basal MHC-I and attenuated IFNγ responsiveness, the proteasome inhibitor bortezomib restored STAT1/pSTAT1 signaling and increased surface MHC-I (with a parallel rise in PD-L1). Analysis of metastatic CRC samples mirrored these findings: STAT1-high tumors exhibited more immunogenic microenvironments, characterized by higher tumor and stromal MHC-I/PD-L1 expression and increased TILs, along with transcriptomic signatures of IFNγ, HLA-A/E/G, and cytotoxic effectors (125). Mechanistically, this places bortezomib→STAT1 priming upstream of checkpoint therapy, and practically it nominates STAT1 as a biomarker to select patients and track on-treatment pharmacodynamics in PI–immune checkpoint inhibitor (ICI) combinations (125).

Yet raising total MHC-I is not sufficient; the peptides shown also change. IFNγ exposure in patient-derived CRC organoids rewired the immunopeptidome through multiple levers—source-protein abundance, induction of the immunoproteasome, and allele-specific increases in presentation (42). Significantly, peptide sequence composition predicted directionality: ligands with proline in their core tended to be downregulated after IFNγ, independent of predicted HLA binding or stability. Thus, when pairing vaccines or TCR therapies with checkpoint blockade in IFNγ-rich niches, epitope selection should incorporate processing constraints, not just binding scores, because the epitope hierarchy can be re-ranked by cytokine signaling (42).

Cytotoxic and DNA-damage–related agents can further enhance visibility when used in conjunction with the appropriate sequence and schedule. With topoisomerase inhibition, blocking NF-κB signaling enhanced MHC-I upregulation, whereas inhibiting ATM/CHK blunted it; moreover, adaptive tolerance to topo blockade reduced both PD-L1 and MHC-I across MSI and MSS lines (127). These data suggest that short lead-in priming with regimens that enhance MHC-I expression is beneficial before initiating anti-PD-1/PD-L1 therapy, while avoiding pathway inhibitors that suppress antigen display during the checkpoint-sensitive window (127).

A complementary, ligandome-expanding approach is to reshape the repertoire itself. The IAP antagonist Birinapant increased the number and abundance of eluted HLA-I ligands and their source proteins *in vitro* (including MCF7 breast-cancer cells) and in xenografts; it enriched CTA-derived peptides, raised neoantigen counts, and provided functional validation of an indel-derived immunogenic epitope (43). Tumors exposed to Birinapant displayed higher immunogenicity, aligning with improved activity when combined with checkpoint inhibitors. Pharmacologically, this supplies not just more ligands but more foreign ones, the ideal substrate for reinvigorated T cells under PD-1/PD-L1 blockade (43).

Checkpoint synergy is, at heart, visibility engineering. A mechanistically grounded framework is to (i) re-arm IFNγ sensing (bortezomib→STAT1) where it is impaired, (ii) choose epitopes with IFNγ-induced processing in mind, (iii) time DNA-damage agents to raise rather than suppress MHC-I, and (iv) expand the ligandome with Birinapant to introduce CTAs and new neoantigens. Prospective trials should embed pre/post-immunopeptidomics, STAT1-centric IHC, and TCR-repertoire tracking alongside clinical response to prove that antigen-presentation remodeling translates into deeper and more durable benefit from checkpoint blockade.

### 5.3 Non-canonical & PTM epitopes

Tumors present antigens that extend well beyond gene-contiguous peptides. Two complementary strata, proteasome-spliced (non-canonical) ligands and post-translationally modified ligands, now have sufficiently rigorous evidence to be taken seriously as vaccine and T-cell therapy targets. A large, method-aware re-analysis of cancer ligandomes reveals that HLA-I proteasome-spliced peptides (PTSPs) constitute a small but consistent subset of the presented repertoire (<3.1%), with HLA-II spliced ligands detectable but rarer (<0.5%) (47). Crucially, many PTSPs were recurrent across samples (including at single-cell–clone resolution) and traced to cancer/immune genes such as MITF, DAPK1, and HLA-E; selected ligands were validated synthetically and shown to be immunogenic, settling earlier debates that had reported inflated prevalence yet preserving the key biological point: splicing creates shared, targetable sequences beyond the linear proteome (47).

In parallel, an updated synthesis of PTM-bearing HLA ligands catalogues ≈approximately 2,450 class-I peptides carrying modifications, including phosphorylation, O-GlcNAcylation, methylation, and kynurenine, accumulated over three decades of MS method development (46). Many of these PTM ligands are disease-biased and observed across multiple patients, a property that supports their candidacy as shared, potentially off-the-shelf targets. Beyond enumeration, the compendium distils practical advances in enrichment, detection, and sequencing that raise confidence in site-localized PTM calls, an essential guardrail when nominating modified peptides for translational programs (46).

Combined, the two datasets redefine target selection. Rare ≠ irrelevant: while PTSPs are relatively rare by count, their absolute count competes with other outlier classes, and recurrency + immunogenicity places them firmly in vaccine/TCR therapy targeting range (47). Second, PTMs can “de-tolerize” the self: phosphorylation- or O-GlcNAc-dependent ligands can avoid central tolerance and emerge preferentially in malignant signaling states, with tumor-biased epitopes and cross-patient penetration (46). Third, validation standards are now clearer: for PTSPs, pipelines should enforce restricted splicing grammars, tailored decoys, and peptide-level FDR, followed by orthogonal verification (synthetic peptides/PRM) and T-cell assays; for PTMs, artefact controls and site localization are mandatory before clinical nomination (46, 47). Finally, these classes are complementary: splicing introduces new sequence order, while PTMs overlay new chemistry on existing sequences, together expanding the antigenic search space in tumors with few classical mutation-derived neoantigens.

A pragmatic path is to (i) screen tumors (or atlases) under a stringent false discovery rate (FDR) for recurrent PTSPs and recurrent post-translationally modified ligands; (ii) verify cell-surface presentation and CD8^+^ reactivity; and (iii) formulate multi-epitope payloads that hedge heterogeneity while tracking on-target pharmacodynamics (TCR tracking, targeted MS re-detection of the exact peptide). Because cytokine signaling and pathway drugs can re-rank epitope hierarchies, embedding pre- and post-immunopeptidomics in early-phase trials will be essential to prove that non-canonical and PTM targeting translates into durable clinical benefits(1, (2).

## 6 Challenges and caveats

Checkpoint drugs and vaccines only work against what tumors actually show; the catch is that presentation is dynamic, context-dependent, and method-sensitive. Longitudinal data already demonstrate that therapeutic perturbations can re-rank targets over clinical timescales. In GBM receiving an oncolytic Herpes Simplex Virus (CAN-3110), repeat biopsies as early as 15–30 days revealed coordinated increases in HLA-I/II and rapid, patient-specific shifts in the immunopeptidome, including a tilt toward cancer-testis antigens, making clear that a single baseline map rarely captures the antigenic state vaccines or T cells will encounter on therapy (160).

Even without intervention, prediction does not equal presentation. In colorectal cancer organoids profiled to deep coverage (~9,936 unique ligands per model), only 3 of 612 non-silent mutations yielded MS-detected class I neoantigens, despite algorithms nominating approximately 304 binders (124). IFNγ exposure increased HLA expression and remodeled the ligandome class II, but did not reveal new class I neoantigens; MEK inhibition likewise failed to do so. These data provide a realistic lower limit (≈0.5%) for mutation-to-ligand mapping in non-hypermutated CRC, sounding the alarm against payloads that are entirely based on in-silico predictions (124). In other words, observe first; predict second.

Nudging translation toward cap-independent initiation (e.g., eIF4A inhibition, silvestrol) can enrich nuORF-derived ligands; conversely, global translation blockade (homoharringtonine) dampens presentation—an avoidable pitfall if vaccines or TCR therapies are dosed into that window (140). DNA-topology drugs are also stealthy: topoisomerase inhibition in some colon cancer models resulted in modest MHC-I increases (≈5%), while others showed significant expansion (≈83%), and 40–50% of eluted peptides were only released upon treatment, with source proteins biased towards extracellular vesicle and nuclear compartments. The implication of timing is preconditioning visibility first, followed by anti-PD-1/PD-L1 delivery second, with pathway inhibitor avoidance (e.g., ATM/CHK) that suppresses MHC-I during the checkpoint-sensitive time window (128).

State-of-the-art low-input workflows (e.g., MHC1-TIP) recover robust HLA-I ligandomes from sub-milligram specimens and even permit parallel proteome and immunopeptidome quantification from the same tissue; yet, primary tumors still show pronounced intratumoral heterogeneity and a weak correlation between protein abundance and peptide display. Translation: neither a single core nor bulk proteomics is a reliable proxy for what is presented. Multi-region sampling, pooled cores, or targeted re-detection (synthetics/PRM) should be budgeted as part of assay design to reduce false negatives/positives (168).

When biology is pushed out of steady state, the risk of over-calling predicted neoantigens that never surface, mistaking drug-induced stress ligands for durable tumor targets, or overlooking regional HLA loss/LOH) increases. At a minimum, pipelines should enforce global and peptide-level FDR, allele deconvolution, and orthogonal validation (synthetic peptide/PRM and T-cell reactivity) before clinical nomination. Trials that embed pre- and post-immunopeptidomics, STAT1-centric pathology when using priming agents, and TCR tracking will be best positioned to prove that visibility remodeling translates into durable benefits. Antigen maps are durable if we honor their temporality, drug dependence, and measurement boundaries. Time spent designing (on-treatment profiling), observation (and not prediction-only) gating material, and drugs programmed to elevate, rather than suppress, display, and orthogonal confirmation convert nice lists into reliable targets.

## 7 Future perspectives: toward a clinical immunopeptidomics roadmap

The following phase is not about discovering more ligands; it is about deriving decisions from observations that can be scaled across patients and will pass regulatory scrutiny. A practical roadmap is emerging: standardize how maps appear and are reported, design for the population first and then personalize, expand the anticipated payload beyond the cancer genome when biology allows, anchor eligibility to biomarkers of antigen visibility, and confirm in treatment that the nominated ligands are actually expressed. Commons that reprocess primary tissues with uniform, auditable pipelines and global/peptide-level FDR demonstrate that harmonized analytics can directly support target nomination and retrospective vaccine design, turning *ad-hoc* maps into a clinical substrate (50). Population reach comes next: interpretable models trained on large ligandomes (e.g., ImmuneApp) advance multi-allelic deconvolution and immunogenicity triage, while resources quantifying presentation promiscuity across alleles, individuals, and populations (CARMEN) enable off-the-shelf panels with maximal HLA coverage onto which patient-specific ligands can be layered (169, 170). Payload diversification should be evidence-led rather than speculative. Microbe-derived peptides are naturally presented and immunogenic in human tumors (CRC, GBM), justifying hybrid payloads (tumor-encoded + microbial) in indications with defined microbial footprints, paired with targeted MS re-detection and on-treatment T-cell functionality as pharmacodynamic readouts (132, 156). Likewise, proteasome-spliced peptides are infrequent but recurrent and immunogenic when identified under splicing-aware decoys and peptide-level FDR with orthogonal validation; under these guardrails, they merit inclusion as curated components of multi-epitope products rather than broad, prediction-only lists (47). Finally, because response to checkpoint and antigen-precise therapies depends on IFNγ sensitivity/antigen-processing, visibility biomarkers (e.g., PSMB9-linked inflamed states in melanoma) should inform who proceeds directly to vaccines/TCRs and who first requires visibility priming; trials should pre-specify on-treatment verification by targeted MS and HLA-aware pathology to ensure that the exact ligands remain displayed when therapy is delivered (50, 75). A disciplined operating system, standardized pipelines, coverage-first design, curated microbial and non-canonical strata, visibility biomarkers, and on-treatment verification are what will enable immunopeptidomics to transition from artisanal discovery to a repeatable, regulator-ready pathway for vaccines and T-cell therapies.

## 8 Technological revolutions enabling immunopeptidome discovery

Over the past five years, a convergent wave of advances spanning ion-mobility hardware, real-time MS acquisition, low-input chemistries, clinical-grade proteogenomics, and population-scale atlases has transformed immunopeptidomics from an exploratory discipline into a platform with direct clinical traction. The common denominator is more depth, higher specificity, less input, and faster turnaround, enabling the discovery of physiologically presented targets (including noncanonical and driver-mutant ligands) and the rational design of vaccines and T-cell therapies. Gas-phase fractionation to see more with less. High-field asymmetric waveform ion-mobility spectrometry (FAIMS) introduced differential ion mobility spectrometry (DIM-MS) into routine immunopeptidomics, enabling the identification of deep ligandomes from small clinical tissues (126). In paired colorectal tumors and normals (mean input ~43 mg), FAIMS-assisted DIM-MS identified 44,815 unique HLA-I ligands and directly detected driver-mutant neoantigens (e.g., KRAS-G12V) with parallel-reaction-monitoring confirmation proof that hardware-level separation increases sensitivity and specificity without prohibitive sample requirements. Methodologically, this narrows the gap between real-world biopsy material and the discovery of actionable ligands (126).

Building on this depth, NeoDiscMS couples next-generation sequencing to real-time spectral acquisition, prioritizing spectra most likely to contain mutated or tumor-associated ligands (171). In the first clinical demonstrations, NeoDiscMS enhanced the detection of tumor-antigen–derived peptides by ~20%, while maintaining global depth and minimizing hands-on complexity. This acquisition-layer solution addresses the long-standing trade-off between depth and sensitivity, offering a practical path toward shorter turnaround times in the clinic (171).

Depth and sensitivity are only as proper as the pipeline that integrates MS with genomics/transcriptomics and prioritizes real targets. NeoDisc addresses this with an end-to-end, clinical pipeline that unifies immunopeptidomics, WES/RNA-seq, and rule-based/ML prioritization across canonical, noncanonical, viral, and high-confidence tumor-specific antigens while simultaneously flagging defects in antigen-processing machinery that shape visibility (172). Head-to-head evaluations demonstrated superior neoantigen prioritization compared to recent tools, operationalizing a “observe-then-predict” paradigm for individualized vaccine design (172). Scale and standardization now come from public repositories. The Ligand.MHC Atlas processed >5,800 immunopeptidome samples (~306 M spectra) to yield ~1.02 M unique ligands (≈583k HLA-I, 435k HLA-II) across 292 HLA alleles and 26 cancers, with ~373k post-translationally modified peptides annotated (49). Batch-effect correction and allele deconvolution enable cross-study comparability, establishing frequency baselines and HLA coverage needed to justify off-the-shelf targets and to benchmark patient-specific hits (49).

NESSIE (Neoantigen selection using a surrogate immunopeptidome) leverages an autologous wild-type immunopeptidome to infer class-I and class-II neoantigens without requiring tumor MS input. In colorectal and endometrial cancers, NESSIE directly identified immunogenic neoepitopes and supported preventive vaccination in a mouse model, broadening access to immunopeptidomics where tumor material is limited or archival (173). MHC1-TIP consolidates enrichment and analysis into a single-tube, cost-effective workflow that recovers robust MHC-I ligandomes from sub-milligram clinical tissues and remains compatible with parallel proteome profiling on the same sample. Application to primary tumors exposed intratumoral heterogeneity in presentation that was poorly correlated with source-protein abundance, empirically underscoring why expression ≠ presentation and why joint proteome–immunopeptidome readouts matter for target triage (168).

The PCI-DB synthesizes >10,000 raw files (>3,000 samples) processed in an equivalent nf-core workflow, and a global FDR, revealing >6.6 M HLA-I peptides and >3.4 M HLA-II peptides across >40 tissue types. Furthermore, PCI-DB went beyond being a discovery vehicle and facilitated the retrospective design of both a TAA-rich and neoepitope-based personalized vaccine that elicited a durable, sustained T-cell response, as well as encouraging outcomes with long-term follow-up, demonstrating how standardized repositories are facilitating translation now (50).

In summary, these technologies create a practical clinical immunopeptidomics stack: (i) hardware/chemistry for depth at low input (FAIMS, single-tube workflows); (ii) intelligent acquisition tuned to the patient mutanome (NeoDiscMS); (iii) end-to-end informatics that coalesce multi-omics to expose APM liabilities (NeoDisc); and (iv) population-scale atlases and surrogate approaches (Ligand, MHC, PCI-DB, NESSIE) to enhance generalizability. A mature field also needs guardrails: consistent global/peptide-level FDR, orthogonal validation (synthetics, PRM), allele-aware deconvolution, and reporting—best practices that these platforms increasingly embody. The net represents a step-change from “predict-and-hope” to “observe-prioritize-validate”, which enables the integration of antigen-precise therapies under real-world clinical constraints.

## 9 Conclusion

The tumor immunopeptidome is not a static fingerprint of antigen processing but a dynamic interface continuously reshaped by tumor-intrinsic pathways and microenvironmental stress. Dysregulation of TAP and ERAP, reprogramming of the proteasome–immunoproteasome axis, and the emergence of post-translationally modified peptides collectively determine whether tumors remain visible or invisible to immune surveillance. Notably, subunit-specific differences highlight that the loss of TAP1 is indicative of profound antigen-presentation failure, and that TAP2 expression level may serve as a prognostic biomarker and a marker for patient selection for immunotherapies that work by enhancing antigen presentation. Importantly, these mechanisms do not merely reflect immune escape but expose therapeutic vulnerabilities: ERAP modulation can recalibrate peptide diversity, immunoproteasome expression can stratify patients for checkpoint therapy, and post-translationally modified-derived peptides represent a new class of shared tumor antigens. Yet, the prognostic and therapeutic value of these pathways is context-dependent, shaped by lineage, inflammatory tone, and metabolic stress within the TME. Since ERAP1 haplotypes classify trimming ability from hypo- to hyper-functional, it is reasonable to consider haplotype-aware patient selection in the use of ERAP1 inhibitors or other ERAP-modulating strategies in immuno-oncology. The next phase of translation will therefore require biomarker-guided strategies that integrate immunopeptidomic profiling with clinical trial design. Framing the immunopeptidome as both a mechanism of immune evasion and a substrate for precision immunotherapy positions it as a central lever in future cancer care.
